# Rare germline variants in *POLE* and *POLD1* encoding the catalytic subunits of DNA polymerases ε and δ in glioma families

**DOI:** 10.1186/s40478-023-01689-5

**Published:** 2023-11-21

**Authors:** Christine A. M. Weber, Nicole Krönke, Valery Volk, Bernd Auber, Alisa Förster, Detlef Trost, Robert Geffers, Majid Esmaeilzadeh, Michael Lalk, Arya Nabavi, Amir Samii, Joachim K. Krauss, Friedrich Feuerhake, Christian Hartmann, Bettina Wiese, Frank Brand, Ruthild G. Weber

**Affiliations:** 1 Department of Human Genetics, Hannover Medical School, Carl-Neuberg-Str. 1, 30625 Hannover, Germany; 2 Department of Neuropathology, Institute of Pathology, Hannover Medical School, Hannover, Germany; 3 Laboratoire CERBA, Saint-Ouen l’Aumône, France; 4 Genome Analytics Research Group, Helmholtz Centre for Infection Research, Braunschweig, Germany; 5 Department of Neurosurgery, Hannover Medical School, Hannover, Germany; 6 Department of Neurosurgery, KRH Klinikum Nordstadt, Hannover, Germany; 7 Department of Neurosurgery, International Neuroscience Institute, Hannover, Germany; 8 Institute for Neuropathology, University Clinic Freiburg, Freiburg, Germany; 9 Department of Neurology, Henriettenstift, Diakovere Krankenhaus gGmbH, Hannover, Germany

**Keywords:** Glioma risk, *POLE*, *POLD1*, Polymerase proofreading defect, Immune checkpoint inhibitors

## Abstract

Pathogenic germline variants in the DNA polymerase genes *POLE* and *POLD1* cause polymerase proofreading-associated polyposis, a dominantly inherited disorder with increased risk of colorectal carcinomas and other tumors. *POLE/POLD1* variants may result in high somatic mutation and neoantigen loads that confer susceptibility to immune checkpoint inhibitors (ICIs). To explore the role of *POLE*/*POLD1* germline variants in glioma predisposition, whole-exome sequencing was applied to leukocyte DNA of glioma patients from 61 tumor families with at least one glioma case each. Rare heterozygous *POLE*/*POLD1* missense variants predicted to be deleterious were identified in glioma patients from 10 (16%) families, co-segregating with the tumor phenotype in families with available DNA from several tumor patients. Glioblastoma patients carrying rare *POLE* variants had a mean overall survival of 21 months. Additionally, germline variants in *POLD1*, located at 19q13.33, were detected in 2/34 (6%) patients with 1p/19q-codeleted oligodendrogliomas, while *POLE* variants were identified in 2/4 (50%) glioblastoma patients with a spinal metastasis. In 13/15 (87%) gliomas from patients carrying *POLE/POLD1* variants, features of defective polymerase proofreading, e.g. hypermutation, *POLE/POLD1*-associated mutational signatures, multinucleated cells, and increased intratumoral T cell response, were observed. In a CRISPR/Cas9-derived *POLE*-deficient LN-229 glioblastoma cell clone, a mutator phenotype and delayed S phase progression were detected compared to wildtype *POLE* cells. Our data provide evidence that rare *POLE/POLD1* germline variants predispose to gliomas that may be susceptible to ICIs. Data compiled here suggest that glioma patients carrying *POLE/POLD1* variants may be recognized by cutaneous manifestations, e.g. café-au-lait macules, and benefit from surveillance colonoscopy.

**Supplementary Information:**

The online version contains supplementary material available at 10.1186/s40478-023-01689-5.

## Introduction

Brain and nervous system tumors account for 1.6% of all newly diagnosed cancer cases worldwide [1]. Adult-type diffuse gliomas comprise IDH-wildtype glioblastomas of CNS WHO grade 4 including rare histological subtypes, such as giant cell glioblastoma or gliosarcoma, IDH-mutant astrocytomas of CNS WHO grade 2, 3, or 4, and IDH-mutant and 1p/19q-codeleted oligodendrogliomas of CNS WHO grade 2 or 3 [2]. Although most gliomas occur sporadically, familial aggregation of glioma cases is observed in around 5% of patients [3, 4]. Moreover, an increased risk of several different brain tumor types is observed in certain tumor syndromes, such as Cowden syndrome associated with meningiomas and gangliocytomas, neurofibromatosis type 2 associated with schwannomas and meningiomas, and familial adenomatous polyposis associated with gliomas and medulloblastomas [5], linking germline variants in *PTEN*, *NF2*, and *APC* to predisposition to a variety of brain tumors. Pathogenic germline variants in other genes, e.g. *POT1* [6] and *CDH1* [7], more specifically increase the risk of a particular brain tumor, namely oligodendroglioma, among other tumors.

Next-generation sequencing has been instrumental in identifying new tumor predisposition syndromes. A decade ago, germline variants in the proofreading domain of *POLE* and *POLD1* were found to predispose to colorectal adenomas and carcinomas, defining a new tumor syndrome, polymerase proofreading-associated polyposis (PPAP) [8]. Since then, other tumors, such as endometrial and ovarian cancer, have been added to the PPAP tumor spectrum [9]. *POLE* and *POLD1* encoding the catalytic subunits of DNA polymerases ε and δ play a central role in suppression of mutagenesis and tumor development by highly accurate DNA replication and exonuclease proofreading [10]. Consequently, *POLE* and *POLD1* have been identified as novel drivers of somatic hypermutation when mutated [11]. Hypermutated colorectal cancer with *POLE/POLD1* variants may benefit from therapy with immune checkpoint inhibitors (ICIs) [12], providing new treatment opportunities for patients carrying *POLE/POLD1* variants.

In this study, we highlight the role of rare heterozygous germline variants in *POLE* and *POLD1* in glioma predisposition and the risk of developing spinal metastases. Features of defective polymerase proofreading in most gliomas from patients carrying *POLE/POLD1* variants suggest that these tumors may be susceptible to ICIs.

## Materials and methods

### Human samples

The study was approved by the Ethics Board of Hannover Medical School. The tumor family cohort comprised 61 tumor families, each with at least one glioma and one other tumor case, including 39 families with at least two brain tumor cases. The index glioma patients underwent brain surgery in Hannover, Germany. In these 61 families, brain tumors were diagnosed in 109 patients: 66 (60.55%) had astrocytic tumors CNS WHO grade 1, 2, 3, or 4, 26 (23.85%) had brain tumors not otherwise specified (NOS), nine (8.3%) had oligodendrogliomas CNS WHO grade 2 or 3, four (3.7%) had meningiomas CNS WHO grade 1 or 2, two (1.8%) had subependymomas CNS WHO grade 1, one (0.9%) had an optic nerve glioma NOS, and one (0.9%) had a glioma NOS. The other tumors included colorectal cancer (21 cases), breast cancer (11 cases), prostate cancer (6 cases), uterus cancer (5 cases), blood cancer (4 cases), gastric cancer (3 cases), lung cancer (3 cases), skin cancer (3 cases), testicular cancer (3 cases), pancreatic cancer (2 cases), and other cancer types. From these 61 tumor families, blood samples from 62 glioma patients were available for genetic testing, including six patients diagnosed with a 1p/19q-codeleted oligodendroglioma. Blood samples from 28 other patients with 1p/19q-codeleted oligodendrogliomas were also available, allowing analysis of leukocyte DNA from a total of 34 patients with a 1p/19q-codeleted oligodendroglioma. One glioblastoma patient (Fam011-III.1/M1) from the tumor family cohort developed a spinal metastasis, and blood or tumor samples of three other glioblastoma patients with a spinal metastasis were analyzed also. Formalin-fixed, paraffin-embedded (FFPE) specimens of 15 gliomas from 11 patients with rare *POLE* or *POLD1* variants, six *POLE* wildtype (WT) gliomas, one *MSH6*-mutated glioma, and one small cell lung cancer were analyzed. Peripheral blood and tumor specimens were subjected to DNA extraction using the QIAamp DNA Blood Maxi Kit (Qiagen, Hilden, Germany) or the QIAamp DNA FFPE Advanced UNG Kit (Qiagen).

### Whole-exome sequencing (WES)

WES was performed on leukocyte DNA of 62 glioma patients from 61 tumor families using the SureSelectXT Human All Exon V5, SureSelectXT Human All Exon V5 + UTRs or SureSelectXT Human All Exon V6 + UTRs target enrichment kit (all Agilent, Santa Clara, CA, USA) on a HiSeq 2500 or NovaSeq 6000 sequencer (both Illumina, San Diego, CA, USA). All samples were sequenced to a mean target coverage of ≥ 50x. Sequencing data were processed and aligned to the GRCh37/hg19 reference human genome assembly using the QIAGEN CLC Genomics Workbench (version 22.0.2; Qiagen), and assessed using Qiagen Clinical Insight Interpret (Qiagen) and our in-house next-generation sequencing data analysis workflow as described in Results and the Additional file 1: Table S1. Variant minor allele frequencies (MAF) were retrieved from the Genome Aggregation Database (gnomAD) browser v2.1.1, controls, non-Finnish European population (https://gnomad.broadinstitute.org). For prediction of variant deleteriousness, the tools MutationTaster (https://www.mutationtaster.org), SIFT (https://sift.bii.a-star.edu.sg), PolyPhen-2 (http://genetics.bwh.harvard.edu/pph2/), PROVEAN (https://www.jcvi.org/research/provean), and CADD (https://cadd.gs.washington.edu) were used.

### Targeted sequencing

Verification of rare *POLE* and *POLD1* variants predicted to be deleterious detected by WES on leukocyte DNA of glioma patients from 10 tumor families was performed by targeted sequencing. Leukocyte DNA of 28 patients with 1p/19q-codeleted oligodendrogliomas not analyzed by WES was screened for variants in all exons of *POLD1* (NCBI reference sequence: NG_033800.1, NM_002691.4). On leukocyte or tumor DNA of three glioblastoma patients who developed spinal metastases (patients M2, M3, and M4), targeted sequencing of all exons of *POLE* (NCBI reference sequence: NG_033840.1, NM_006231.4) was performed. Amplicons generated using customized oligonucleotides (Additional file 1: Table S2) and standard molecular techniques were sequenced using conventional chain termination protocols on a 3130xl Genetic Analyzer (Thermo Fisher Scientific, Waltham, MA, USA) or by GATC Services (Eurofins Scientific, Luxemburg, Luxemburg). All non-silent variants were assessed with respect to MAF and pathogenicity, as described above.

### Tumor mutational burden (TMB) and mutational signature analysis

Tumor DNA extracted from 14 FFPE glioma specimens from 10 patients with *POLE* or *POLD1* variants was sequenced using xGen customized gene panels (Integrated DNA Technologies, Coralville, IO, USA) targeting cancer-associated genes on a NextSeq 500 sequencer (Illumina) to a mean target coverage of 185x. Sequencing data were processed and aligned to the GRCh37/hg19 reference human genome assembly, and the TMB score of each glioma DNA was calculated using the QIAGEN CLC Genomics Workbench. Variants from the dbSNP common database (dbSNP common v151 ensembl hg19), which contains variants with a MAF ≥ 0.01, were subtracted to obtain somatic variants. To calculate TMB, the total number of somatic non-synonymous high-quality (read depth ≥ 10, call quality ≥ 50, allele fraction ≥ 5%) single nucleotide variants in the coding region was divided by the total size of the sequenced coding region in megabases. To detect combinations of mutation types within the identified somatic coding non-synonymous single nucleotide variants of each glioma DNA, the Mutational Signatures in Cancer (MuSiCa) tool (http://bioinfo.ciberehd.org/GPtoCRC/en/tools.html), an R-based web application to characterize mutational signatures in cancer samples based on COSMIC database v2 - March 2015 [13], was used. The nomenclature of the detected mutational signatures was updated to single base substitution (SBS) signatures of COSMIC database v3.3.

### Histological assessment

Hematoxylin and eosin staining of sections of 15 FFPE gliomas from 11 patients with *POLE* or *POLD1* variants was performed according to standard protocols. Sections were evaluated by an experienced neuropathologist (CH) regarding the presence of enlarged nuclei or multinucleated cells. Images were acquired using a BX46 microscope and an XC50 camera (all Olympus, Shinjuku, Japan), or sections were scanned using an Aperio AT2 Scanner (Leica Microsystems, Wetzlar, Germany), and digital images were processed using Aperio ImageScope v12.4.3.5008 software (Leica Microsystems).

### Multiplexed fluorescence immunohistochemistry (IHC)

To characterize the immune cell infiltrate of seven glioblastomas and two spinal metastases from seven patients with *POLE* variants as well as *POLE* WT glioblastomas and a spinal metastasis, multiplexed fluorescence IHC was performed on 3 μm thick FFPE glioma sections. OPAL Multiplex IHC Assay kit (Akoya Biosciences, Marlborough, MA, USA) was used for spectral library generation and data acquisition following the manufacturer’s instructions (OPAL Multiplex IHC Assay Development Guide, Akoya Biosciences). Slides were deparaffinized, initial antigen retrieval was performed by microwave cooking in Tris-buffered saline at pH 9.0, and blocking of unspecific protein binding was performed using Protein Block Serum-free solution (Agilent). Subsequent antigen retrieval and deactivation of the preceding staining step were performed by microwave cooking either in Tris-buffered saline at pH 9.0 or citrate buffer at pH 6.0. Consecutive IHC staining using the OPAL 9-plex fluorescence system was performed using the following primary antibodies: anti-CD3 (clone A0452, Agilent; dilution 1:200), anti-CD4 (clone SP35, Zytomed Systems, Berlin, Germany; dilution 1:50), anti-CD8 (clone C8/144B, Agilent; dilution 1:200), anti-CD34 (QBEnd/10, Leica Microsystems; dilution 1:200), anti-CD68 (clone PGM-1, Agilent; dilution 1:750), anti-PD-1 (clone NAT105, Merck, Darmstadt, Germany; dilution 1:100), anti-PD-L1 (clone QR1, Quartett, Berlin, Germany; dilution 1:200), anti-GFAP (6F2, Agilent; dilution 1:400). Nuclear staining was performed by 4′,6-diamidino-2-phenylindole (DAPI) (Akoya Biosciences). The following fluorophores in combination with the tyramide signal amplification system were used for detection of bound antibodies: Opal 480, Opal 520, Opal 540, Opal 570, Opal 620, Opal 650, Opal 690 or Opal 780. Fluoromount-G mounting medium (Thermo Fisher Scientific) was applied to cover slides before imaging. PD-L1 staining was performed using chromogenic duplex-IHC according to standard staining protocols on an automated staining instrument (VENTANA BenchMark ULTRA, Roche, Basel, Switzerland).

### Multispectral imaging and quantitative evaluation

Fluorescent whole slide image scanning was performed at 20x magnification using the PhenoImager HT instrument (Akoya Biosciences). Whole slide images were used to select fields of view (FOV) that cover all tumor areas excluding necrosis and artifacts (i.e. tissue folding, disruption, hemorrhages etc.) for subsequent targeted scanning of image stacks at 40x magnification across the visible spectrum (440–780 nm). For each tumor, 19–151 FOV were scanned. Spectral libraries were generated using single-stained scans of tonsil tissue for each reagent, and multispectral color deconvolution was performed with the inForm image analysis software (inForm v2.6, Akoya Biosciences). Quantitative evaluation of CD3, CD4, CD8, CD68, and PD-1 expression in immune cells was performed on a total of 1122 FOV using an automated analysis algorithm (inForm) and R packages phenoptr (R package version 0.3.2, https://akoyabio.github.io/phenoptr/) and phenoptrReports (https://github.com/akoyabio/phenoptrReports/). Visual quality control of all analyzed FOV was performed by reviewing all composite images in the context of the quantitative output. GFAP and CD34 were used to provide orientation and biological context. PD-L1 was assessed semiquantitatively by visual inspection and grading into positive (> 30% stained tumor cells) and negative tumors.

### Cell culture and transfection

LN-229 glioblastoma cells were cultured in high-glucose Dulbecco’s Modified Eagle Medium (Merck) supplemented with 10% fetal bovine serum, 2 mM L-glutamine, 1% penicillin/streptomycin (all Thermo Fisher Scientific). For HCT116 colorectal cancer cells, RPMI 1640 medium (Merck) supplemented with 10% fetal bovine serum, 1% penicillin/streptomycin was used. Transient transfection was done using Lipofectamine 3000 reagent (Thermo Fisher Scientific).

### Multicolor fluorescence *in situ* hybridization (FISH) analysis

To determine the karyotype of LN-229 glioblastoma cells, 24-color FISH [14] was performed on metaphase chromosome preparations using the 24XCyte Human Multicolor FISH Probe Kit (MetaSystems, Altlussheim, Germany), which contains probes for all human chromosomes labeled with specific fluorochrome combinations, according to the manufacturer’s protocol. Fluorescent images were captured with a Leica DCX epifluorescence microscope (Leica Microsystems) coupled to a charge-coupled device camera (Teledyne Photometrics, Tucson, AZ, U.S.A.) and equipped with appropriate filter sets to allow specific detection of each fluorochrome used. Multicolor FISH data were processed using the Leica CW4000 software (Leica Microsystems).

### CRISPR/Cas9-mediated editing of *POLE* and *POLD1*

Editing of *POLE* and *POLD1* in LN-229 and HCT116 cells was done using a protocol for CRISPR/Cas9-mediated RNA-guided genome editing [15]. Single guide RNA (sgRNA) sequences targeting *POLE* exon 2 or *POLD1* exon 3 were designed using the CRISPOR web-based tool (http://www.crispor.tefor.net), along with sense and antisense oligonucleotides (Additional file 1: Table S2). The synthesized sgRNA was inserted by T4 DNA ligase into a *Bpi*I-digested *pSpCas9(BB)-2A-GFP* plasmid (#48138, Addgene, Watertown, MA, USA) containing an sgRNA scaffold and expression cassettes for Cas9 and GFP. By transient transfection, the Cas9/sgRNA construct was introduced into LN-229 and HCT116 cells. After 24 h, GFP-positive cells were isolated using a FACSAria Fusion Flow Cytometer (Becton, Dickinson and Company, Franklin Lakes, NJ, USA). After cell expansion, their genomic DNA was extracted using the innuPREP DNA Mini Kit (Analytik Jena, Jena, Germany). To identify the genotype of selected cell clones, polymerase chain reaction products of *POLE* exon 2 and *POLD1* exon 3 were analyzed by direct sequencing. In selected cell clones used for cellular assays, exonic sgRNA off-target sites adjacent to a PAM site were analyzed by direct sequencing (primer sequences are listed in the Additional file 1: Table S2).

### Western blot analysis

POLE expression was analyzed in selected edited LN-229 cell clones. After sodium dodecylsulfate-polyacrylamide gel electrophoresis and semidry electroblotting, polyvinylidene difluoride membranes (General Electric, Boston, MA, USA) were treated with 5% fat-free milk powder in phosphate-buffered saline with 0.05% Tween 20 (PBST) as blocking agent. The following primary antibodies were diluted in 5% (w/v) BSA in PBST and used for immunodetection: mouse anti-POLE (MABE966, clone 9F11.1, Merck; dilution 1:1,000) and rabbit anti-β-tubulin (#2128, 9F3, Cell Signaling Technology, Danvers, MA, USA; dilution 1:1,000). After incubation overnight at 4 °C, membranes were exposed to the appropriate horseradish peroxidase-conjugated secondary antibody (donkey anti-mouse, #A16017 or donkey anti-rabbit, #A16035, both Thermo Fisher Scientific; dilution 1:2,000) in 5% fat-free milk powder in PBST for 60 min at room temperature, and developed using the SuperSignal West Dura Extended Duration Substrate (Thermo Fisher Scientific). Signals were acquired using the Fusion FX7 gel documentation system (Vilber, Collégien, France).

### Flow cytometry-based cell cycle analysis

To analyze cell cycle progression in selected edited LN-229 cell clones, the FITC BrdU Flow Kit (Becton, Dickinson and Company) was used. For each cell line, 0.4 × 10^6^ cells were seeded in 60 mm culture dishes and grown for two days. Cells were pulse-labeled with 10 µM 5-bromo-2′-deoxyuridine (BrdU) in culture medium for 30 min to label newly synthesized DNA, washed twice with medium and cultivated further. After 0, 4, 8, and 10 h, cells were washed with phosphate-buffered saline, harvested by scraping from plates, and fixed with BD Cytofix/Cytoperm Buffer (Becton, Dickinson and Company). Fixed cells were stored overnight at 4 °C and pretreated with DNase for 1 h to expose BrdU epitopes before staining with fluorescein-5-isothiocyanat (FITC)-conjugated anti-BrdU antibody followed by total DNA staining with 7-aminoactinomycin D (7-AAD). Cells were measured on a BD FACSLyric Flow Cytometry System (Becton, Dickinson and Company), and data were analyzed using FlowJo software (v10.8.0; Becton, Dickinson and Company).

### Hypoxanthine phosphoribosyltransferase 1 (*HPRT1*) mutation assay

The mutation rate was determined in selected edited LN-229 cell clones using a *HPRT1* mutation assay. Briefly, cells were grown for 9 days (passaged every 2–3 days) in medium containing 100 µM hypoxanthine, 0.4 µM aminopterin and 16 µM thymidine (HAT, Thermo Fisher Scientific) to remove pre-existing 6-thioguanine (6-TG) resistant cells. After HAT treatment, cells were recovered for 3 passages, and 10^4^ cells per well were seeded in 48-well plates. After 24 h, cells were incubated with 0.5 µM, 5 µM, 50 µM 6-TG (Cayman Chemical, Ann Arbor, MI, USA) dissolved in growth medium, or, as control, in medium without 6-TG, and cultivated for 5 more days. The medium was changed after 3 days. Cells were simultaneously fixed and stained by incubation with 4% (m/v) paraformaldehyde in phosphate-buffered saline, 5% (v/v) methanol, and 0.5% (m/v) crystal violet (Sigma-Aldrich, St. Louis, MO, USA) for 30 min. Subsequently, culture plates were carefully immersed in water to remove the fixation and staining mixture, and dried overnight at room temperature before staining was documented. For quantification of the crystal violet staining intensity, grayscale images acquired using the Fusion FX7 gel documentation system (Vilber) were analyzed using Fiji/ImageJ software (version 1.52n, https://imagej.nih.gov/ij/).

### Statistical analysis

Data are presented as mean and standard deviation. Statistical significance was calculated using two-tailed Student’s *t* test, whereby *p* values of ≤ 0.05 (*), and ≤ 0.01 (**) were considered statistically significant.

## Results

### Rare heterozygous germline variants in the PPAP genes *POLE* and *POLD1* were detected in 10/61 (16%) tumor families with at least one glioma case each

**Fig. 1:**
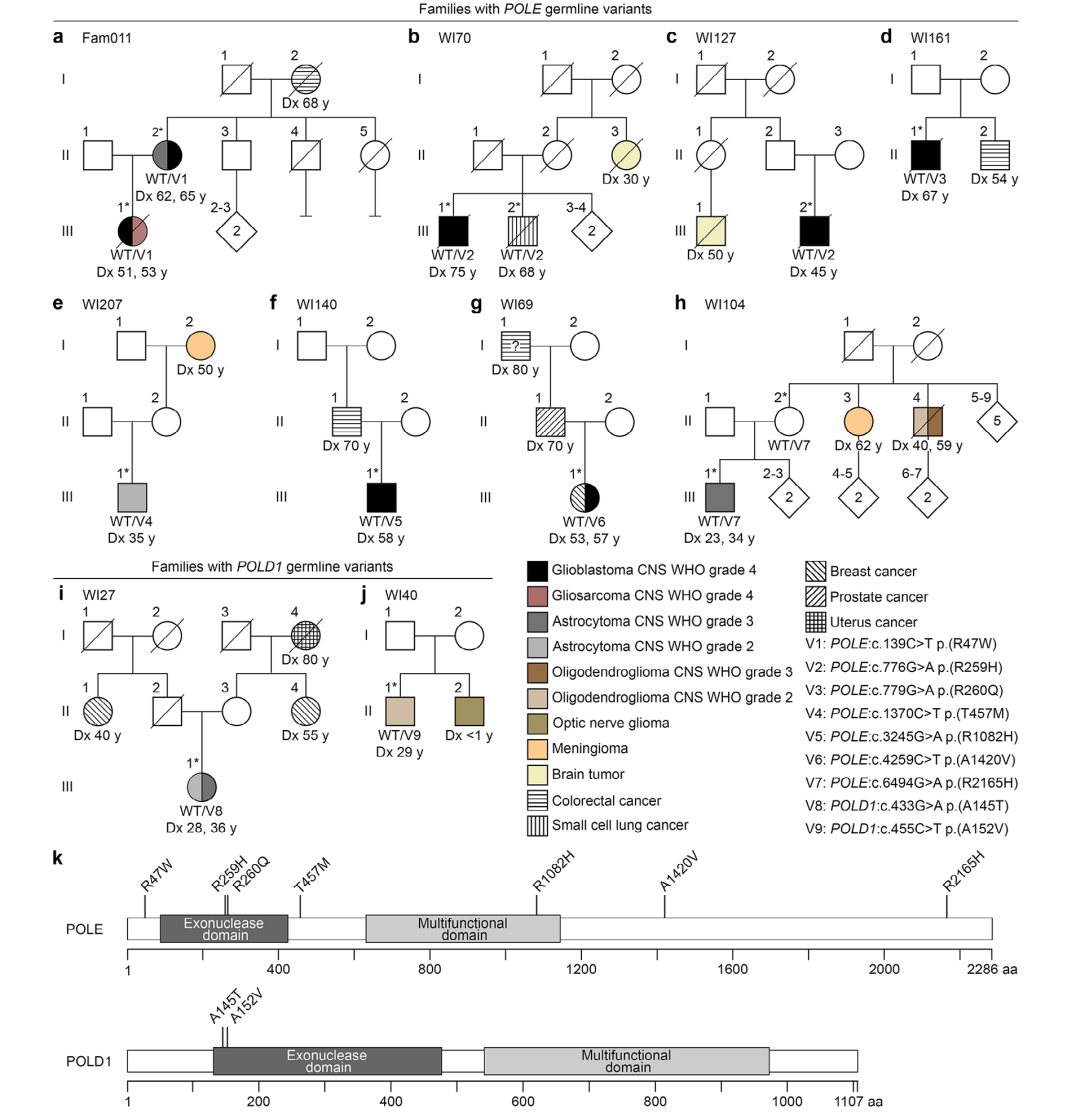
Pedigrees of 10 tumor families with at least one glioma case each carrying rare heterozygous *POLE* (**a-h**) or *POLD1* (**i-j**) germline missense variants, and variant localization (**k**). (**a**) Co-segregation of *POLE* variant V1 with the glioma phenotype in patients III.1/M1 and II.2 of family Fam011. Patient I.2 was diagnosed with colorectal cancer. (**b**) Co-segregation of *POLE* variant V2 with the tumor phenotype in glioblastoma patient III.1 and small cell lung cancer patient III.2 of family WI70. (**c**) The *POLE* variant V2 was also identified in glioblastoma patient III.2 of family WI127. (**d**) In family WI161, glioblastoma patient II.1 carries the *POLE* variant V3. Patient II.2 was diagnosed with colorectal cancer. (**e**) In family WI207, patient III.1 with an astrocytoma CNS WHO grade 2 carries the *POLE* variant V4. Patient I.2 was diagnosed with meningioma. (**f**) In family WI140, glioblastoma patient III.1 carries the *POLE* variant V5. Patient II.1 was diagnosed with colorectal cancer. (**g**) The *POLE* variant V6 was identified in patient III.1 of family WI69 who was diagnosed with breast cancer at age 53 years and glioblastoma at age 57 years. (**h**) In family WI104, patient III.1 with an astrocytoma CNS WHO grade 3 carries the *POLE* variant V7. Patient II.3 was diagnosed with meningioma, and patient II.4 with oligodendroglioma. (**i**) In family WI27, astrocytoma patient III.1 carries the *POLD1* variant V8. Patients II.1 and II.4 were diagnosed with breast cancer, and patient I.4 with uterus cancer. (**j**) In family WI40, patient II.1 with an oligodendroglioma CNS WHO grade 2 carries the *POLD1* variant V9. Patient II.2 was diagnosed with optic nerve glioma. (**k**) Domain structures of the catalytic subunit of DNA polymerase ε (POLE) and the catalytic subunit of DNA polymerase δ (POLD1), and localization of the identified *POLE/POLD1* variants. Exonuclease domain (IPR006133) and multifunctional domain (IPR006134) of POLE/POLD1 according to the InterPro database (https://www.ebi.ac.uk/interpro/). Aa, amino acids; Dx, age at diagnosis; CNS, central nervous system; V, variant; WHO, World Health Organization; WT, wildtype; y, years. Asterisks indicate patients of whom DNA was available for genetic testing. A question mark indicates that the tumor diagnosis is uncertain

Using WES and a linkage-based strategy (Additional file 1: Table S1), the *POLE*:c.139C>T p.(R47W) variant was identified as the only rare (MAF ≤ 0.01) deleterious germline variant, not present in controls and located in a cancer predisposition gene, co-segregating with the glioma phenotype in index tumor family Fam011 with two glioma patients and a colorectal carcinoma patient in three consecutive generations (Fig. 1a; Table 1). The *POLE* variant was verified to be heterozygous by targeted sequencing in both glioma patients (Additional file 2: Fig. S1).

**Table 1:** Rare *POLE* and *POLD1* variants predicted to be deleterious identified in this study

Patient ID	Gene	Genomic position (GRCh37/hg19)	Nucleotide change	Amino acid change	dbSNP^a^	MAF^b^	Prediction according to				CADD score^g^	Previously described in germline of patients with
							MutationTaster^c^	SIFT^d^	Poly Phen-2^e^	PROVEAN^f^		
**Heterozygous** ***POLE*** **and** ***POLD1*** **germline variants identified in glioma patients from 10 tumor families**												
Fam011-III.1/M1	*POLE*	12:133257789	c.139C>T	p.(R47W)	rs143626223	0.001243	DC	D	PrD	Del	27.1	BC [9], CRC [9, 16–18], EC [19], OC [9]
Fam011-II.2												
WI70-III.1	*POLE*	12:133253974	c.776G>A	p.(R259H)	rs61732929	0.009734	DC	T	B	N	23.3	BC [9, 20], CRC [9, 20], OC [9]
WI127-III.2												
WI161-II.1	*POLE*	12:133253971	c.779G>A	p.(R260Q)	rs5744752	0.0001242	DC	D	B	Del	23.8	BC [9], CRC [9]
WI207-III.1	*POLE*	12:133249853	c.1370C>T	p.(T457M)	rs878854842	-	DC	D	PoD	Del	26.1	CRC [21], PTC [22]
WI140-III.1	*POLE*	12:133235911	c.3245G>A	p.(R1082H)	rs201744227	0.0002486	DC	T	B	Del	25.5	BC [9], CRC [17, 23]
WI69-III.1	*POLE*	12:133220454	c.4259C>T	p.(A1420V)	rs41561818	0.004536	DC	T	B	N	21.2	BC [9, 24], CC [24], CRC [9, 25], Mel [24], OC [9]
WI104-III.1	*POLE*	12:133202740	c.6494G>A	p.(R2165H)	rs5745068	0.003258	DC	T	PrD	Del	25.9	BC [9, 24, 26], CC [24], CRC [9, 27], EC [28], Mel [24], OC [9]
WI27-III.1	*POLD1*	19:50905151	c.433G>A	p.(A145T)	rs137953986	0.002778	DC	T	B	N	20.4	BC [24], CC [24], Mel [24]
WI40-II.1	*POLD1*	19:50905173	c.455C>T	p.(A152V)	rs41563714	0.001057	DC	T	B	N	20.5	BC [9], CRC [29], Mel [9], OC [9]
**Heterozygous** ***POLE*** **variant identified in the glioblastoma of a patient who developed a spinal metastasis**												
M2^h^	*POLE*	12:133201381	c.6763A>T	p.(I2255F)	rs73155056	0.005655	DC	T	B	N	22.3	BC [9], CRC [9], OC [9]
**Homozygous** ***POLD1*** **germline variant identified in an oligodendroglioma patient without family history of cancer**												
WII-40-II.1	*POLD1*	19:50918229	c.2546G>A	p.(R849H)	rs3218775	0.008146	DC	T	B	N	22.1	CRC [30]

Given are all identified rare (minor allele frequency, MAF ≤ 0.01), non-silent (i.e. splice site, frameshift, in-frame indels, stop gained/lost and non-synonymous missense) variants predicted to be deleterious by at least one prediction tool, i.e. MutationTaster, SIFT, PolyPhen-2 or PROVEAN, in the *POLE* or *POLD1* gene. NCBI reference sequence NM_006231.4 (*POLE*) and NM_002691.4 (*POLD1*). B, benign; BC, breast cancer; CC, cervical cancer; CRC, colorectal cancer; D, damaging; DC, disease causing; Del, deleterious; EC, endometrial cancer; Mel, melanoma; N, neutral; OC, ovarian cancer; PoD, possibly damaging; PTC, papillary thyroid cancer; PrD, probably damaging; T, tolerated

^a^SNP database ID (https://www.ncbi.nlm.nih.gov/SNP/)

^b^According to the Genome Aggregation Database (gnomAD) browser v2.1.1, controls, non-Finnish European population (https://gnomad.broadinstitute.org)

^c^According to MutationTaster (https://www.mutationtaster.org)

^d^According to SIFT (https://sift.bii.a-star.edu.sg)

^e^According to PolyPhen-2 (http://genetics.bwh.harvard.edu/pph2/)

^f^According to PROVEAN (https://www.jcvi.org/research/provean)

^g^According to CADD (https://cadd.gs.washington.edu)

^h^Only tumor DNA of the patient was analyzed (non-neoplastic DNA was not available)

This finding prompted us to investigate the role of rare variants in the *POLE* and *POLD1* genes in glioma risk. Taken together, seven different rare *POLE* and two different rare *POLD1* variants, heterozygous and predicted to be deleterious, were detected in WES datasets of leukocyte DNA of a total of 11 glioma patients from 10 of 61 (16%) tumor families, including the glioma patients from family Fam011 (Fig. 1a-j, Additional file 2: Fig. S1, Table 1). The CADD score of all variants was ≥ 20, indicating that they are considered to be among the top 1% most deleterious variants in the human genome (Table 1). All variants were previously described in the germline of patients with colorectal cancer, breast cancer, ovarian cancer, endometrial cancer, cervical cancer, melanoma or other tumors (Table 1). Three *POLE* variants affected amino acids located within known functional protein domains of POLE, two within the exonuclease domain, i.e. c.776G>A p.(R259H) and c.779G>A p.(R260Q), and one within the multifunctional domain, i.e. c.3245G>A p.(R1082H) (Fig. 1k). Both *POLD1* variants, i.e. c.433G>A p.(A145T) and c.455C>T p.(A152V), affected amino acids located within the exonuclease domain of POLD1 (Fig. 1k).

In both families with available DNA from two tumor patients each, the rare *POLE* variants, i.e. c.139C>T p.(R47W) in family Fam011 and c.776G>A p.(R259H) in family WI70, co-segregated with the tumor phenotype (Fig. 1a, b). The *POLE*:c.776G>A p.(R259H) variant was identified in a total of three patients from two families who were affected by glioblastomas CNS WHO grade 4 or small cell lung cancer (Fig. 1b, c). The *POLE*:c.4259C>T p.(A1420V) variant was detected in a patient with a glioblastoma CNS WHO grade 4 who had had breast cancer four years earlier (Fig. 1g). The *POLE*:c.6494G>A p.(R2165H) variant was identified in a patient with astrocytoma CNS WHO grade 3 that had recurred after 11 years, and in his tumor-unaffected mother whose siblings were affected by a meningioma CNS WHO grade 2 or an oligodendroglioma CNS WHO grade 2 that had recurred as an oligodendroglioma CNS WHO grade 3 after 19 years (Fig. 1h).

Glioma patients carrying rare *POLE*/*POLD1* variants were primarily affected by glioblastoma CNS WHO grade 4 (6/11, 55%), astrocytoma CNS WHO grade 3 (2/11, 18%), astrocytoma CNS WHO grade 2 (2/11, 18%), and oligodendroglioma CNS WHO grade 2 (1/11, 9%) at a mean age of 48 years (range: 23–75 years), and with a mean overall survival of 59 months (range: 2-165 months) (Fig. 1; Table 2). When considering only the six *POLE/POLD1* variant carriers with a glioblastoma CNS WHO grade 4, their mean age was 59 years and their mean overall survival was 21 months. Six of 10 (60%) families carrying *POLE/POLD1* variants had at least two family members with brain tumors (Fig. 1a-c, e, h, j; Table 2), with gliomas occurring together with meningiomas in two families (Fig. 1e, h). An optic nerve glioma had been diagnosed at under one year of age in the sibling of a *POLD1* variant carrier (Fig. 1j). In the 10 families carrying *POLE/POLD1* variants, other tumors were colorectal cancer (4/10, 40%), breast cancer (2/10, 20%), uterus cancer (1/10, 10%), prostate cancer (1/10, 10%), and small cell lung cancer (1/10, 10%) (Fig. 1; Table 2).

**Table 2:** Clinical characteristics of glioma patients carrying rare *POLE* or *POLD1* variants predicted to be deleterious

Patient ID	Gene	Amino acid change	Gender	Age at Dx^a^	P/ R/ M	Localization	Histology	CNS WHO grade	Molecular characteristics	Overall survival^b^	Other tumors in patient/family
**Glioma patients from 10 tumor families carrying rare** ***POLE*** **or** ***POLD1*** **germline variants**											
Fam011-III.1/M1	*POLE*	p.(R47W)	Female	51	P	Right insula	Glioblastoma	4	IDH-WT	20	CRC
				53	M	Spinal cord	Gliosarcoma	4	IDH-WT		
Fam011-II.2			Female	62	P	Left FT	Astrocytoma	3	-	137^d^	
				65	R	Left FT	Glioblastoma	4	-		
WI70-III.1	*POLE*	p.(R259H)	Male	75	P	Right FP	Glioblastoma	4	IDH-WT	14	Brain tumor NOS, SCLC
WI127-III.2			Male	45	P	Left FP	Glioblastoma	4	IDH-WT	26	Brain tumor NOS
WI161-II.1	*POLE*	p.(R260Q)	Male	67	P	Left frontal	Glioblastoma	4	IDH-WT	18	CRC
WI207-III.1	*POLE*	p.(T457M)	Male	35	P	Right parietal	Astrocytoma	2	IDH-mut	2^d^	Meningioma NOS
WI140-III.1	*POLE*	p.(R1082H)	Male	58	P	Left basal ganglia	Glioblastoma	4	IDH-WT	36^d^	CRC
WI69-III.1	*POLE*	p.(A1420V)	Female	57	P	Left temporal	Glioblastoma	4	IDH-WT	12^d^	BC, PC, CRC?
WI104-III.1	*POLE*	p.(R2165H)	Male	23	P	Left frontal	Astrocytoma	3	-	157^d^	Meningioma CNS WHO grade 2, oligodendroglioma CNS WHO grade 2 and 3
				34	R	Left frontal	Astrocytoma	3	IDH-mut		
WI27-III.1	*POLD1*	p.(A145T)	Female	28	P	Right central	Astrocytoma	2	-	165^d^	BC, UC
				36	R	Right frontal	Astrocytoma	3	IDH-mut		
WI40-II.1	*POLD1*	p.(A152V)	Male	29	P	Left frontal	Oligodendroglioma	2	IDH-mut, 1p/19q-codel	60^d^	Optic nerve glioma
**Glioblastoma with a rare** ***POLE*** **variant from a patient who developed a spinal metastasis**											
M2^c^	*POLE*	p.(I2255F)	Male	62	P	Right temporal	Glioblastoma	4	IDH-WT	8	NA
				63	M	Spinal cord	Gliosarcoma	4	IDH-WT		
**Oligodendroglioma patient without family history of cancer carrying a rare** ***POLD1*** **variant**											
WII-40-II.1	*POLD1*	p.(R849H)	Female	34	P	Right frontal	Oligodendroglioma	3	IDH-mut, 1p/19q-codel	3^d^	-

NCBI reference sequence NM_006231.4 (*POLE*) and NM_002691.4 (*POLD1*). BC, breast cancer; CNS, central nervous system; codel, codeleted; CRC, colorectal cancer; Dx, diagnosis; FP, frontoparietal; FT, frontotemporal; M, metastasis; mut, mutated; NA, not available; NOS, not otherwise specified; P, primary tumor; PC, prostate cancer; R, recurrent tumor; SCLC, small cell lung cancer; UC, uterus cancer; WHO, World Health Organization; WT, wildtype

^a^Years

^b^Months

^c^The *POLE* variant was detected in the primary tumor and the spinal metastasis of the patient (non-neoplastic DNA was not available)

^d^Until last follow up

### Rare *POLD1* germline variants were identified in 2/34 (6%) oligodendroglioma patients

As one of the two *POLD1* germline variants was detected in a patient with an oligodendroglioma (Fig. 1j), a rare brain tumor characterized by a 1p/19q codeletion [2] comprising the *POLD1* locus at 19q13.33, we hypothesized that *POLD1* germline variants may be particularly frequent in patients with oligodendrogliomas. Therefore, we performed *POLD1* mutational analysis on leukocyte DNA of 33 other oligodendroglioma patients. We identified the rare homozygous *POLD1*:c.2546G>A p.(R849H) missense variant predicted to be deleterious affecting amino acids located within the multifunctional domain of POLD1 in one sporadic oligodendroglioma patient (Tables 1 and 2, Additional file 2: Fig. S2). Taken together, rare deleterious *POLD1* germline variants were detected in two of 34 (6%) oligodendroglioma patients (Additional file 2: Fig. S2).

### Rare *POLE* variants were observed in 2/4 (50%) glioblastoma patients with spinal metastases

**Fig. 2:**
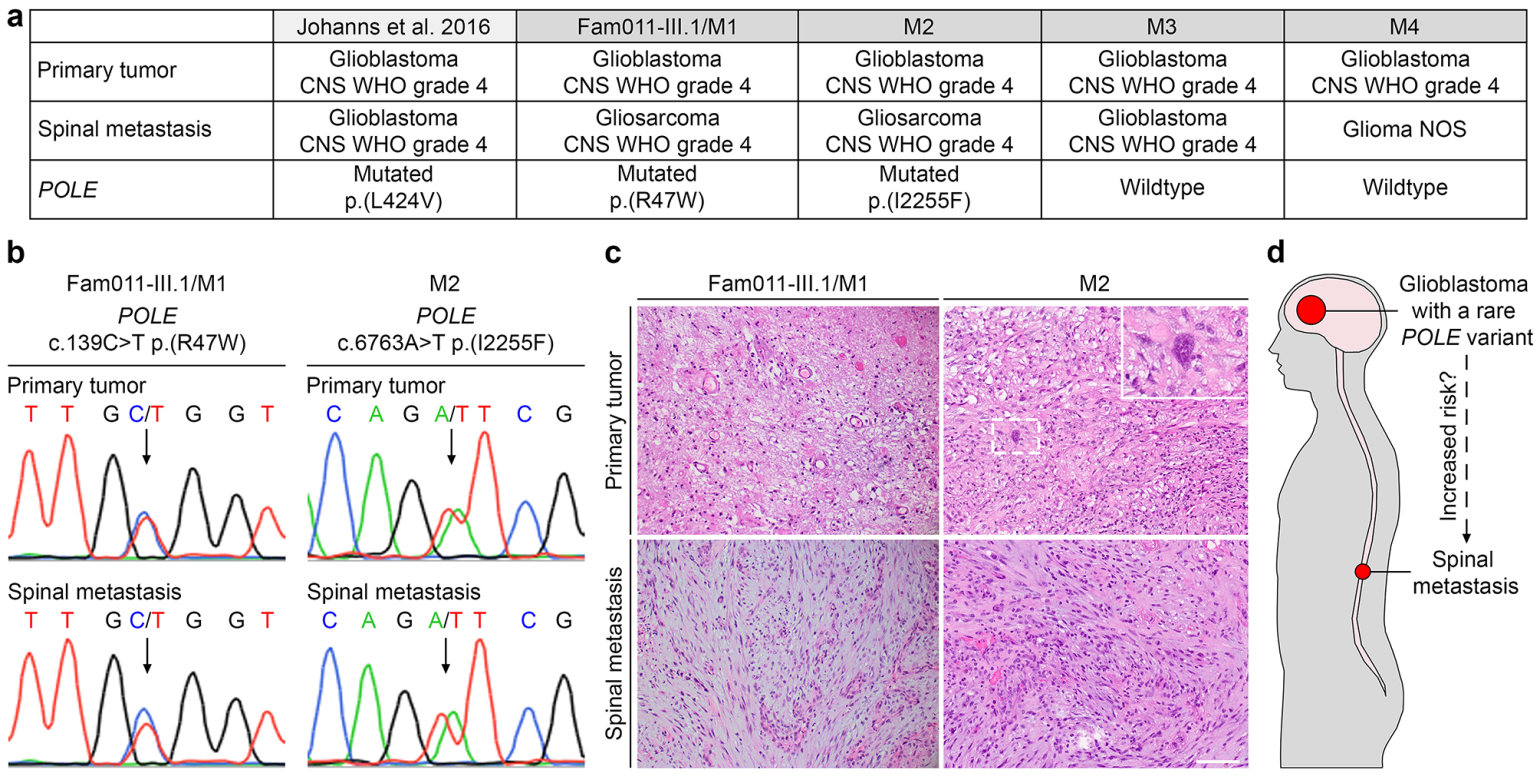
Rare *POLE* variants were detected in glioblastoma patients who developed spinal metastases. (**a**) Summary of the histology and *POLE* variant status of four glioblastoma patients with spinal metastases from this study (patients Fam011-III.1/M1, M2, M3, and M4), and a previously described case [31]. (**b**) Electropherograms showing the *POLE* variants detected in DNA of the glioblastomas and spinal metastases of patients Fam011-III.1/M1 and M2. The position of the variant is indicated by an arrow. (**c**) Hematoxylin and eosin stained sections of the primary tumors diagnosed as glioblastomas, and spinal metastases diagnosed as gliosarcomas of patients Fam011-III.1/M1 and M2. The inset shows a cell with an enlarged nucleus. Scale bar, 30 μm. (**d**) Scheme illustrating our hypothesis that *POLE* variants in glioblastomas may promote the development of spinal metastases. CNS, central nervous system; WHO, World Health Organization; NOS, not otherwise specified

As a glioblastoma patient from our index family (patient Fam011-III.1/M1) and a glioblastoma patient previously described [31], both carrying rare *POLE* germline variants, developed spinal metastases, we performed *POLE* mutational analysis in three other patients with glioblastoma CNS WHO grade 4 and spinal metastasis (patients M2-M4, Fig. 2a). In DNA of both glioblastoma and spinal metastasis of patient M2 (DNA from non-neoplastic tissue was not available), we identified the rare *POLE*:c.6763A>T p.(I2255F) variant that was predicted to be deleterious (Fig. 2b; Tables 1 and 2). The spinal metastases of patients Fam011-III.1/M1 and M2 were both diagnosed as gliosarcoma CNS WHO grade 4 (Fig. 2c; Table 2). Taken together, rare *POLE* variants were identified in two of four (50%) glioblastoma patients who developed spinal metastases. Despite the small number of patients, data from us and others suggest an increased risk of spinal metastases in glioblastomas with rare *POLE* variants (Fig. 2d).

### Features of defective polymerase proofreading, i.e. high TMB, presence of *POLE/POLD1* variant-associated mutational signatures or multinucleated cells/enlarged nuclei, and increased immune cell infiltrate, were detected in 13/15 (87%) gliomas from patients carrying *POLE/POLD1* variants

Primary and recurrent gliomas as well as spinal metastases of patients with rare *POLE/POLD1* variants were assessed with respect to features of polymerase proofreading defects by determining TMB and signatures of somatic mutations in glioma DNA as well as presence of multinucleated cells or enlarged nuclei and immune cell response in glioma sections.

**Fig. 3:**
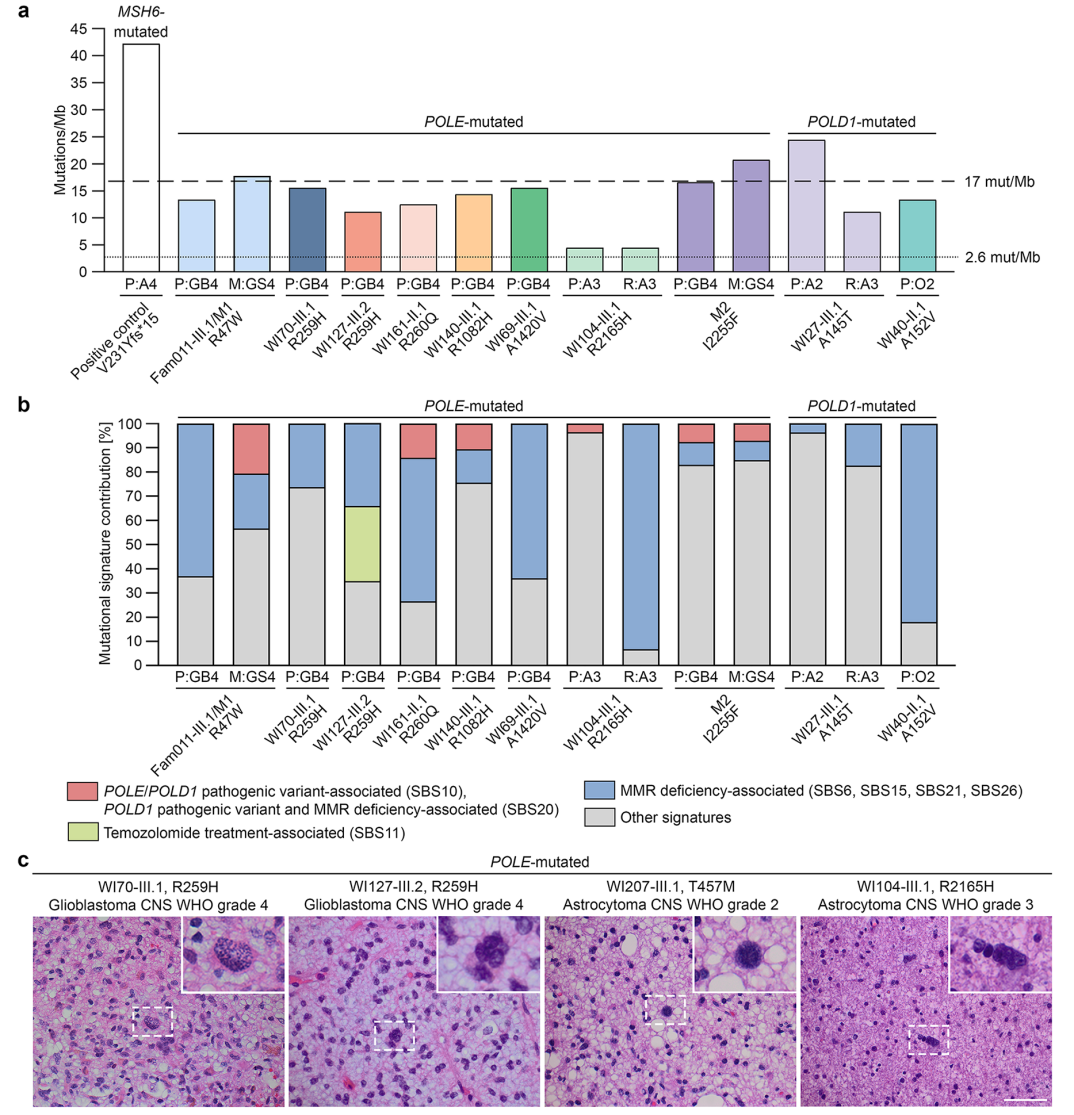
Characterization of gliomas from patients with rare *POLE/POLD1* germline variants with respect to features of defective polymerase proofreading by determining burden and signatures of somatic mutations as well as presence of multinucleated cells or enlarged nuclei. (**a**) Graph showing the tumor mutational burden (TMB), defined as the number of somatic coding non-synonymous mutations per megabase of coding DNA sequence (mut/Mb), of 14 gliomas from 10 patients with rare *POLE/POLD1* variants. The threshold for hypermutation of 17 mut/Mb and the median TMB per glioma of 2.6 mut/Mb, as previously defined for gliomas [32], are shown as dashed or dotted lines. An astrocytoma CNS WHO grade 4 of a patient carrying a homozygous *MSH6* germline frameshift variant served as positive control. (**b**) Graph showing the COSMIC database SBS signatures of somatic mutations of 14 gliomas from 10 patients with rare *POLE/POLD1* germline variants, highlighting the contribution of *POLE/POLD1* pathogenic variant and *POLD1* pathogenic variant and MMR deficiency-associated signatures SBS10 and SBS20, MMR deficiency-associated signatures SBS6, SBS15, SBS21, and SBS26, and temozolomide treatment-associated signature SBS11. (**c**) Hematoxylin and eosin stained sections of four gliomas from patients with rare *POLE* germline variants showing enlarged nuclei (WI70-III.1 and WI207-III.1) or multinucleated cells (WI127-III.2 and WI104-III.1). Scale bar, 60 μm. A2/3/4, astrocytoma CNS WHO grade 2/3/4; CNS, central nervous system; GB4, glioblastoma CNS WHO grade 4; GS4, gliosarcoma CNS WHO grade 4; M, spinal metastasis; MMR, mismatch repair; O2, oligodendroglioma CNS WHO grade 2; P, primary tumor; R, recurrent tumor; WHO, World Health Organization

A mean TMB of 13.9 mutations per megabase (mut/Mb) was identified in 14 glioma DNAs from 10 patients with rare *POLE/POLD1* variants, with each TMB value higher than 2.6 mut/Mb, the median TMB in a study of 10,294 gliomas [32] (Fig. 3a). Three of 14 (21%) *POLE/POLD1*-mutated gliomas, including the spinal metastases of patients Fam011-III.1/M1 (17.8 mut/Mb) and M2 (20.7 mut/Mb), and the primary astrocytoma CNS WHO grade 2 of patient WI27-III.1 (24.4 mut/Mb), had a TMB ≥ 17 mut/Mb and were hypermutated according to Touat et al. 2020 [32] (Fig. 3a). An astrocytoma CNS WHO grade 4 of a patient carrying a homozygous *MSH6* germline frameshift variant, i.e. NM_000179.3(*MSH6*):c.691del p.(V231Yfs*15), had a TMB of 42.2 mut/Mb and served as positive control (Fig. 3a).

While DNA mismatch repair (MMR) deficiency-associated mutational signatures SBS6, SBS15, SBS21, or SBS26 were identified in 13 of 14 (93%) glioma DNAs from 10 patients with rare *POLE/POLD1* variants, a substantial (> 50%) contribution was detected in only 5 of 14 (36%) gliomas (Fig. 3b). *POLE/POLD1* pathogenic variant or *POLD1* pathogenic variant and MMR deficiency-associated mutational signatures SBS10 and SBS20 were detected in 6/14 (43%) cases, including the hypermutated spinal metastases of patients Fam011-III.1/M1 (contribution of mutational signatures SBS10/SBS20: 21%) and patient M2 (7%), and the primary gliomas of patients WI161-II.1 (14%), WI140-III.1 (11%), WI104-III.1 (4%), and M2 (8%) (Fig. 3b). While two of the six gliomas with mutational signatures SBS10/SBS20 were from carriers of *POLE* variants located within or close to the exonuclease domain, four gliomas harbored *POLE* non-exonuclease domain variants. The mutational signature SBS14 associated with concurrent *POLE* pathogenic variant and defective MMR was not observed. The temozolomide treatment-dependent mutational signature SBS11 was not detected in the analyzed recurrent tumors or spinal metastases suggesting that the detected mutational signatures were not therapy-associated.

As defects in polymerase proofreading have been associated with the formation of multinucleated giant cells [33], hematoxylin and eosin stained sections of 15 gliomas from 11 patients with rare *POLE/POLD1* variants were evaluated by an experienced neuropathologist (CH). Enlarged nuclei or multinucleated cells were identified in 6/15 (40%) gliomas from patients with *POLE*/*POLD1* variants, including a primary astrocytoma CNS WHO grade 2, a primary astrocytoma CNS WHO grade 3 and its recurrent tumor of the same grade, and three primary glioblastomas CNS WHO grade 4, two of which carried the *POLE*:c.776G>A p.(R259H) variant (Figs. 2c and 3c, Additional file 1: Table S3).

**Fig. 4:**
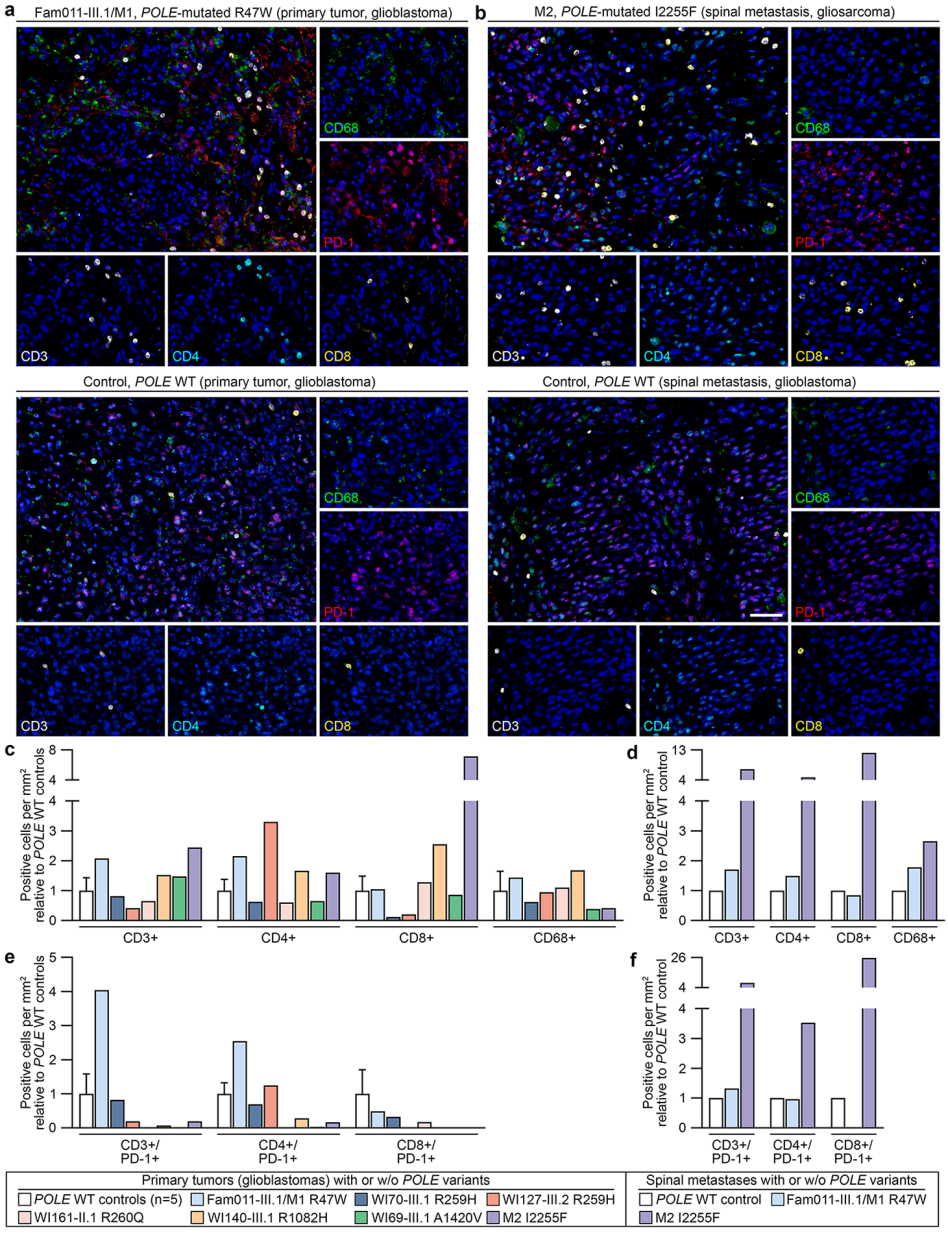
Immune cell composition in *POLE-*mutated compared to *POLE* WT primary glioblastomas and spinal metastases as determined by multiplexed fluorescence immunohistochemistry. (**a**, **b**) Representative images showing immune cells expressing CD3 (white), CD4 (cyan), CD8 (yellow), CD68 (green), PD-1 (red) in sections of *POLE*-mutated and *POLE* WT glioblastomas (**a**) and spinal metastases (**b**) showing an increased immune cell infiltrate in the *POLE*-mutated glioblastoma and spinal metastasis. Nuclei were stained with 4′,6-diamidino-2-phenylindole (DAPI, blue) and used for cell detection. Scale bar, 50 μm. (**c**, **d**) Number of CD3+, CD4+, CD8+ T lymphocytes and CD68+ macrophages per mm^2^ in *POLE*-mutated glioblastomas relative to the mean in five *POLE* WT glioblastomas (**c**), and in *POLE*-mutated spinal metastases relative to a *POLE* WT spinal metastasis (**d**). In all but one *POLE*-mutated glioblastoma or spinal metastasis, the density of total CD3+ T lymphocytes and/or CD4+ or CD8+ subsets was increased compared to controls with WT *POLE*. (**e**, **f**) Number of PD-1-positive CD3+, CD4+, and CD8+ T lymphocytes per mm^2^ in *POLE*-mutated glioblastomas relative to the mean in five *POLE* WT glioblastomas (**e**), and in *POLE*-mutated spinal metastases relative to a *POLE* WT spinal metastasis (**f**). The density of PD-1-positive CD3+ T lymphocytes and/or CD4+ or CD8+ subsets was increased in one or two *POLE*-mutated glioblastomas and spinal metastases compared to controls with WT *POLE*. W/o, without; WT, wildtype

The immune cell infiltrate of seven primary glioblastomas and two spinal metastases from seven patients with rare *POLE* variants as determined by multiplexed fluorescence IHC of immune cell markers CD3, CD4, CD8, CD68, and PD-1 was compared to that in *POLE* WT glioblastomas. The primary glioblastoma of patient Fam011-III.1/M1 showed an increased immune infiltrate compared to a *POLE* WT glioblastoma, which was composed mainly of PD-1-positive CD3+ T lymphocytes co-expressing CD4, and CD68+ macrophages (Fig. 4a, c, e, Additional file 1: Table S3). In the spinal metastasis of patient M2, a gliosarcoma, the increased immune cell infiltrate compared to a *POLE* WT spinal metastasis was composed mainly of PD-1-positive CD3+ T lymphocytes co-expressing CD8 (Fig. 4b, d, f, Additional file 1: Table S3). The immune cell composition was similar in the primary glioblastoma and the spinal metastasis of each patient (Fig. 4, Additional file 1: Table S3). Immune cells were diffusely distributed within the tumor microenvironment. Inclusion of the vascular endothelial marker CD34 in the multiplex panel confirmed that immune infiltrates were not restricted to the perivascular compartment (Additional file 2: Fig. S3). The density of total CD3+ T lymphocytes and/or CD4+ or CD8+ subsets was increased in six of seven (86%) *POLE*-mutated primary glioblastomas, and that of CD68+ macrophages in three of seven (43%) *POLE*-mutated primary glioblastomas compared to the mean density in five *POLE* WT glioblastomas (Fig. 4c, Additional file 1: Table S3). The primary glioblastomas of patients M2 and WI140-III.1, both characterized by increased immune cell infiltration, were positive for PD-L1, i.e. PD-L1 expression was observed in > 30% of tumor cells, as confirmed by chromogenic duplex-IHC staining (Additional file 2: Fig. S4). An increased density of CD3+ T lymphocytes and/or CD4+ or CD8+ subsets and of CD68+ macrophages was detected in both *POLE*-mutated spinal metastases compared to the density in a *POLE* WT spinal metastasis (Fig. 4d). Taken together, in eight of nine (89%) *POLE*-mutated primary glioblastomas or spinal metastases, including three tumors harboring variants classified as likely pathogenic (Additional file 1: Table S4), the density of total CD3+ T lymphocytes and/or CD4+ or CD8+ subsets was increased compared to controls with WT *POLE* (Additional file 1: Table S3).

In summary, at least one feature of defective polymerase proofreading, i.e. presence of hypermutation, *POLE/POLD1* variant-associated mutational signature SBS10 or SBS20, multinucleated cells/enlarged nuclei, and increased infiltrate of T lymphocytes and/or macrophages, was detected in 13/15 (87%) gliomas from *POLE/POLD1* variant carriers compared to controls with WT *POLE* by multimodal assessment (Additional file 1: Table S3), with an impact on the classification of the majority of *POLE/POLD1* variants identified here (Additional file 1: Table S4).

### Delayed S phase progression and a mutator phenotype were identified in a LN-229 glioblastoma cell clone with *POLE* deficiency

**Fig. 5:**
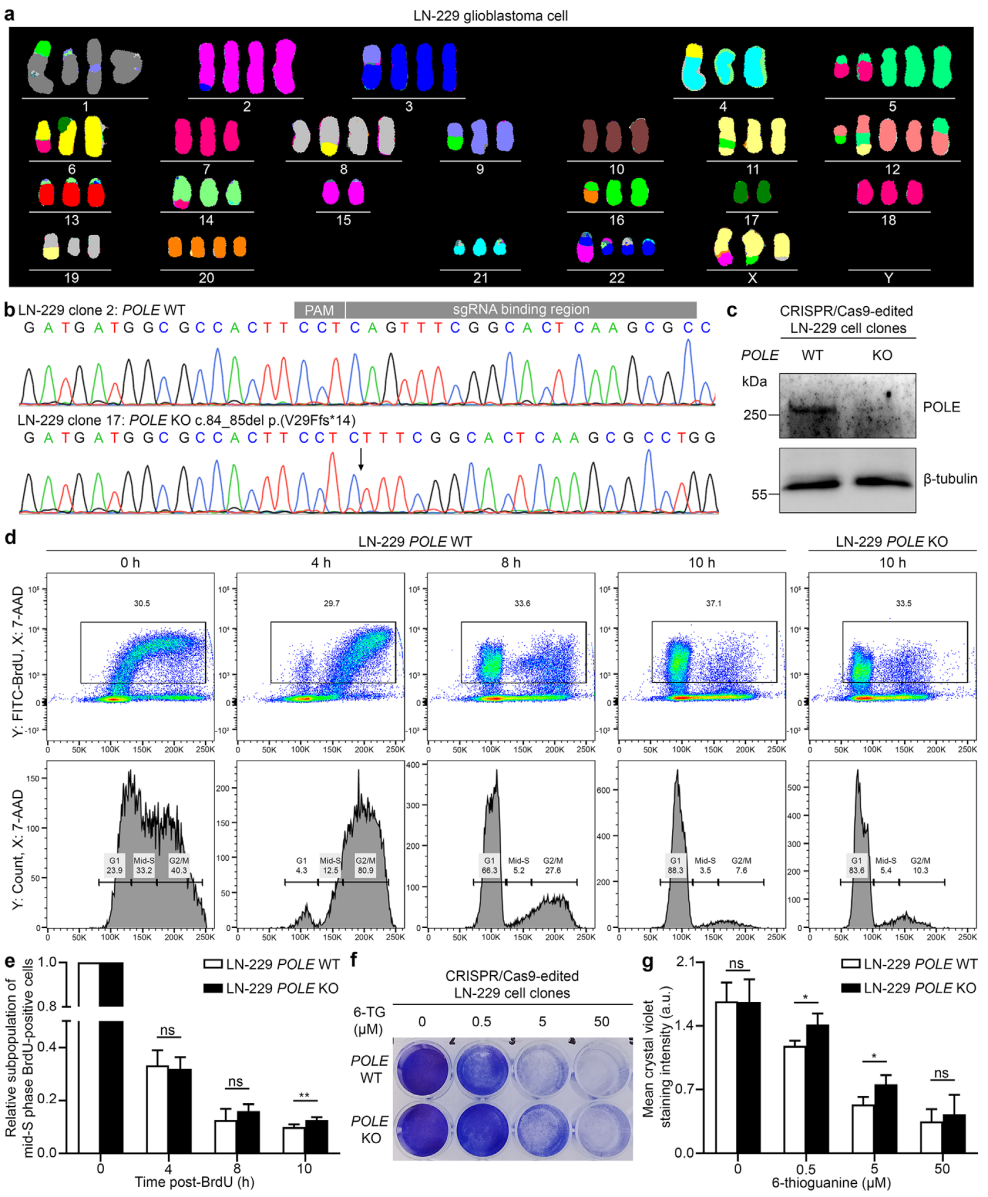
Generation and characterization of a cellular model of *POLE* deficiency in LN-229 glioblastoma cells. (**a**) Multicolor fluorescence in situ hybridization analysis of a metaphase chromosome preparation from an LN-229 glioblastoma cell. In the hyperdiploid karyotype, the long arm of chromosome 12 where *POLE* is localized (12q24.33), and the long arm of chromosome 19 where *POLD1* is localized (19q13.33) are probably present in three copies. (**b**) Electropherograms of the sequence of the sgRNA on-target site in exon 2 of *POLE*. A clone with no mutational event at the sgRNA on-target site (clone 2, *POLE* WT) was selected as control. A single clone was devoid of WT *POLE* alleles and exclusively harbored the *POLE*:c.84_85del p.(V29Ffs*14) frameshift variant at the sgRNA on-target site (clone 17, *POLE* KO). The position of the deletion is indicated by an arrow. (**c**) Western blot analysis of LN-229 *POLE* WT and KO cell lysates using anti-POLE and anti-β-tubulin antibodies. No full-length POLE was detected in the *POLE* KO clone. (**d**) Representative flow cytometry plots and histograms of BrdU pulse-labeled and 7-AAD stained LN-229 *POLE* WT and KO cells at different time points after BrdU treatment. (**e**) Quantification of the mid-S phase BrdU-positive LN-229 *POLE* WT and KO cell subpopulations relative to the value 0 h after BrdU treatment showing a significant increase in *POLE* KO cells 10 h after BrdU compared to *POLE* WT cells suggesting delayed S phase progression. Given are mean and standard deviation of three independent experiments. (**f**) Representative image of a *HPRT1* mutation assay using LN-229 *POLE* WT and KO cells treated with 6-TG for 5 days and stained with crystal violet. (**g**) Quantification of grayscale images of the *HPRT1* mutation assay showing a significantly increased mean crystal violet staining intensity indicating resistance after treatment with 0.5 and 5 µM 6-TG in LN-229 *POLE* KO compared to WT cells suggesting a higher mutation rate. Given are mean and standard deviation of three independent experiments. 7-AAD, 7-aminoactinomycin D; BrdU, 5-bromo-2′-deoxyuridine; KO, knockout; PAM, protospacer adjacent motif; sgRNA, single guide RNA; 6-TG, 6-thioguanine; WT, wildtype. *, p ≤ 0.05; **, p ≤ 0.01; two-tailed Student’s *t* test

LN-229 cells, a glioblastoma cell line, and HCT116 cells, a colorectal cancer cell line devoid of WT *MLH1* alleles [34], were used as cellular models. Multicolor FISH analysis of LN-229 glioblastoma cells revealed a hyperdiploid karyotype with more than two copies of the long arms of chromosome 12 harboring the *POLE* gene at 12q24.33 and possibly of chromosome 19 harboring the *POLD1* gene at 19q13.33 (Fig. 5a). A CRISPR/Cas9-based protocol was used to edit *POLE* and *POLD1* in both cell lines. In LN-229 glioblastoma cells, genotyping of the CRISPR/Cas9-edited cells revealed 1/48 (2%) cell clones harboring a mutant *POLE* allele, 1/48 (2%) cell clones devoid of WT *POLE* alleles, 20/85 (24%) cell clones harboring a mutant *POLD1* allele, and 0/85 cell clones devoid of WT *POLD1* alleles (Additional file 1: Table S5). Using HCT116 cells, no cell clone with a mutant *POLE* or *POLD1* allele or devoid of WT *POLE* or *POLD1* alleles was detected in 37 or 45 cell clones analyzed (Additional file 1: Table S5).

For subsequent analyses, we selected a LN-229 glioblastoma clone with no indel at the *POLE* sgRNA on-target site (clone 2, *POLE* WT) and the only LN-229 glioblastoma clone devoid of WT *POLE* alleles (clone 17, *POLE* KO) shown to exclusively harbor the *POLE*:c.84_85del p.(V29Ffs*14) frameshift variant at the sgRNA on-target site (Fig. 5b) and not to express full-length POLE by Western blot analysis (Fig. 5c). In these two LN-229 glioblastoma clones, no indels were detected in two predicted exonic off-target sites adjacent to a PAM site.

As deficiency of POLE, the catalytic subunit of the major leading-strand DNA polymerase ε, is expected to impact DNA replication, we performed flow cytometry-based cell cycle analysis of BrdU pulse-labeled and 7-AAD-stained *POLE* WT and KO LN-229 glioblastoma cells. At 10 h after BrdU treatment, the relative subpopulation of mid-S phase BrdU-positive cells was significantly increased in *POLE* KO versus WT cells (two-tailed Student’s *t* test, p = 0.0073), suggesting delayed S phase progression in *POLE* KO LN-229 glioblastoma cells (Fig. 5d, e).

To assess whether POLE deficiency affects mutation rate in glioblastoma cells, we performed a *HPRT1* mutation assay in *POLE* WT and KO LN-229 glioblastoma cells. Cells carrying *HPRT1* mutations become resistant to 6-TG, and can be visualized using crystal violet dye after 6-TG treatment. After 5 days of incubation with 0.5 and 5.0 µM 6-TG, the intensity of crystal violet staining was significantly increased in *POLE* KO compared to WT LN-229 glioblastoma cells (two-tailed Student’s *t* test, p = 0.040 and p = 0.044), indicating more cells with 6-TG resistance, i.e. a higher *HPRT1* mutation rate, among *POLE* KO cells (Fig. 5f, g).

Taken together, the viability of LN-229 glioblastoma and MMR-deficient HCT116 cells carrying CRISPR/Cas9*-*mediated *POLE* or *POLD1* variants was markedly reduced. The only viable *POLE* KO LN-229 glioblastoma clone was characterized by impaired S phase progression and a mutator phenotype compared to a *POLE* WT LN-229 glioblastoma clone.

### Brain tumor patients with rare *POLE*/*POLD1* germline variants are frequently affected by giant cell glioblastoma in addition to gastrointestinal and cutaneous phenotypes

**Fig. 6:**
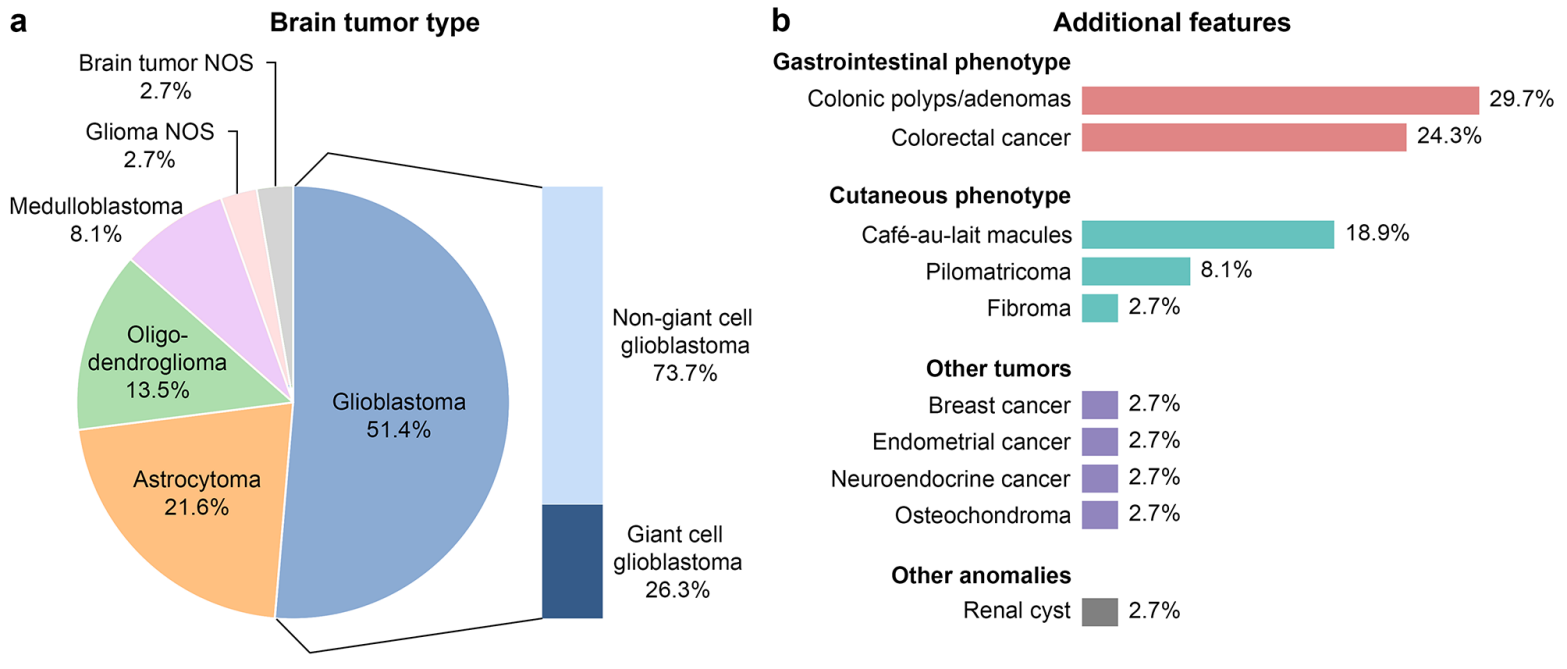
Clinical features of 37 brain tumor patients carrying rare *POLE* or *POLD1* germline variants reported in this study or previously (patients and references are listed in the Additional file 1: Table S6). (**a**) Types of brain tumors observed in patients with rare *POLE*/*POLD1* germline variants. Giant cell glioblastomas are overrepresented. (**b**) Additional features reported in brain tumor patients with rare *POLE*/*POLD1* germline variants. Gastrointestinal and cutaneous phenotypes are particularly frequent. NOS, not otherwise specified

To elucidate the largely unknown phenotype spectrum of brain tumor patients carrying rare *POLE* or *POLD1* germline variants, tumor types and additional features of 37 brain tumor patients with rare *POLE*/*POLD1* germline variants from this study and the literature were compiled. Patients and references are listed in the Additional file 1: Table S6. Of these 37 patients, 19 were diagnosed with glioblastoma (51.4%), eight with astrocytoma (21.6%), five with oligodendroglioma (13.5%), three with medulloblastoma (8.1%), and one each with glioma NOS (2.7%) or brain tumor NOS (2.7%) (Fig. 6a). Thus, the brain tumors in *POLE*/*POLD1* variant carriers with a definitive diagnosis were gliomas or medulloblastomas. Of the 19 glioblastoma patients, 5 (26.3%) were affected by giant cell glioblastoma. Additional features observed in the 37 patients include gastrointestinal and cutaneous phenotypes, such as colonic polyps/adenomas in 11 (29.7%) cases, colorectal cancer in nine (24.3%) cases, café-au-lait macules in seven (18.9%) cases, pilomatricoma in three (8.1%) cases, fibromas in one (2.7%) case, and other tumors, such as breast cancer, endometrial cancer, neuroendocrine carcinoma, and osteochondroma in one (2.7%) case each (Fig. 6b).

## Discussion

Studying tumor families with at least one glioma case each by whole-exome sequencing, rare deleterious *POLE* or *POLD1* germline missense variants were identified in 10 of 61 (16%) families, and were shown to co-segregate with the tumor phenotype in two families with available DNA from two tumor patients. At least two brain tumors were observed in six of the 10 (60%) families with rare *POLE* or *POLD1* germline variants. These data provide evidence that brain tumors, particularly gliomas, are part of the tumor spectrum of PPAP caused by rare *POLE* and *POLD1* germline variants. Similarly, a recent whole-genome sequencing study on families with two glioma cases each identified *POLE/POLD1* germline nonsense variants in glioma patients from three of 189 (1.6%) families of the exploratory cohort [35], although it is controversial whether *POLE/POLD1* loss-of-function variants are pathogenic for PPAP [9]. In line with our findings, a recent report summarizing 132 carriers of probably pathogenic *POLE/POLD1* germline exonuclease domain variants identified 10 (8%) brain tumor cases among these patients [36]. Furthermore, non-benign *POLE* and *POLD1* variants were identified in tumor DNA of 5.3% (*POLE*) or 2.5% (*POLD1*) glioblastomas [37]. Glioblastoma was the most common brain tumor type that was diagnosed in around 50% of the 37 brain tumor patients with rare *POLE*/*POLD1* germline variants compiled here from our data and previous reports (references are listed in the Additional file 1: Table S6). The development of oligodendroglioma may be particularly favored by *POLD1* germline variants, as oligodendrogliomas are characterized by a 1p/19q codeletion affecting the *POLD1* locus at 19q13.33. And indeed, we detected rare *POLD1* germline variants in two of 34 (6%) oligodendroglioma patients. In our summary of 37 brain tumor patients carrying rare *POLE*/*POLD1* germline variants, oligodendrogliomas were found in as many as 13.5% of cases.

In accordance with the tumor spectrum initially [8] and most commonly [36] described in PPAP patients, we observed colorectal cancer in four of 10 (40%) families with rare *POLE*/*POLD1* germline variants. All but one *POLE* or *POLD1* germline variant identified here in glioma patients was previously detected in the germline of patients with colorectal cancer (references listed in Table 2). In the 37 brain tumor patients carrying rare *POLE/POLD1* germline variants compiled here, gastrointestinal phenotypes were the most frequent additional features with colorectal adenomas in around 30%, and colorectal cancer in around 25% of cases. Together, these data imply that regular colonoscopy may be advisable in carriers of rare *POLE/POLD1* germline variants, as recently recommended [36]. These data also suggest that alterations in certain genes may predispose to both gastrointestinal and brain tumors. In addition to *POLE/POLD1* variants, these include *CDH1* aberrations causing hereditary diffuse gastric cancer syndrome that may also increase the risk of brain tumors of neuroepithelial and epithelial origin [7], and variants in the MMR genes *MLH1*, *MSH2*, *MSH6*, or *PMS2* causing constitutional MMR deficiency or hereditary non-polyposis colorectal cancer (or Lynch) syndrome when mutated in a biallelic or heterozygous fashion that are associated with an increased risk of colorectal and brain tumors [38].

Polymerase proofreading, also called DNA replication repair, and MMR are determinants governing DNA replication fidelity, and errors that escape proofreading are corrected by MMR [39]. Pathogenic variants in *POLE* or *POLD1* causing defective polymerase proofreading, and pathogenic variants in MMR genes causing defects in MMR can lead to a high TMB, i.e. hypermutation, in the DNA of human tumors, including brain tumors, when they occur in the germline or somatically [9, 11, 31, 33, 40–42]. *POLE* and *POLD1* germline variants affecting the exonuclease domain required for polymerase proofreading, e.g. *POLE* p.(L424V) and *POLD1* p.(S478N), were initially reported to predispose to colorectal adenomas and carcinomas [8], and some of these variants, e.g. *POLE* p.(P286R), p.(V411L), and p.(L424V), and *POLD1* p.(C319Y), have been experimentally associated with hypermutation [43]. While six of the nine different *POLE* and *POLD1* germline missense variants identified here in glioma patients affect amino acids located within or close to the exonuclease domain, three variants affect amino acids in other parts of POLE. In line with increasing evidence that variants outside of the exonuclease domain can also cause hypermutation [11, 44, 45], a TMB higher than 2.6 mut/Mb, the median of 10,294 gliomas according to Touat et al. [32], was detected in all 14 glioma DNAs from 10 *POLE/POLD1* variant carriers analyzed here, three of which were definitely hypermutated (TMB > 17 mut/Mb [32]). Alternatively, in recurrent gliomas hypermutation may be a consequence of temozolomide treatment [32, 46]. Depending on the cause of hypermutation, e.g. defective polymerase proofreading, MMR defects, or drug treatment, the signature of somatic mutations, i.e. their substitution class and nucleotide context, may differ [47, 48]. Tumors with pathogenic *POLE* or *POLD1* variants, particularly in the exonuclease domain, exhibit characteristic mutational signatures, i.e. COSMIC database signatures SBS10, SBS14, and SBS20 [47, 49]. While mutational signature SBS14 was previously detected in a hypermutated oligodendroglioma of a patient with *de novo POLE* exonuclease variant p.(L424V) [9], mutational signatures SBS10 or SBS20 were found in six of 14 (43%) gliomas, two of which were hypermutated, from patients with rare *POLE/POLD1* variants here, providing evidence for defective polymerase proofreading in these tumors. Similar to the results obtained on tumors from *POLE/POLD1* variant carriers by Mur et al. [9], MMR deficiency-associated mutational signatures substantially contributed to the mutational signature spectrum in 5 of 14 (36%) gliomas analyzed here.

By causing a high TMB, *POLE* variants may also promote the development of spinal metastases. This may explain why we identified rare *POLE* variants in two of four (50%) glioblastoma patients who developed spinal metastases, and another case was reported previously [31], whereas spinal metastases normally develop in only around 1% of glioblastoma patients [50]. We presume that defective polymerase proofreading was the mechanism underlying metastasis formation in both glioblastoma patients carrying *POLE* variants here because the mutational signatures detected in the spinal metastases were associated with *POLE*/*POLD1* pathogenic variants, not with temozolomide. Both spinal metastases were diagnosed as gliosarcoma, a rare subtype of glioblastoma with a similar outcome [51] that is characterized by mixed glial and mesenchymal histopathological features [2], and showed a hypermutator phenotype. Hypermutation as well as germline and somatic variants in MMR genes were recently observed in the spinal metastasis, also a gliosarcoma, of a patient with a low-grade glioma [52], suggesting that defects in both polymerase proofreading and MMR may promote hypermutation, metastasis to the spinal cord, and gliosarcoma development.

Defects in polymerase proofreading and MMR may also be linked to the formation of multinucleated giant cells, possibly also via hypermutation. Consistent with our finding of enlarged nuclei or multinucleated cells in 6/15 (40%) gliomas from patients with *POLE*/*POLD1* germline variants, multinucleated giant or bizarre cells, features of giant cell glioblastoma, were previously observed in high-grade gliomas harboring somatic *POLE* variants [33]. Similarly, around 25% of glioblastoma patients with rare *POLE*/*POLD1* germline variants compiled here were affected by giant cell glioblastoma, although this tumor type typically accounts for < 1% of all glioblastomas [53, 54]. Giant cell enrichment was also observed in glioblastomas with somatic variants in MMR genes [55], suggesting that the giant cell phenotype may point to an underlying defect in DNA replication fidelity.

*POLE* variants may elicit an intratumoral T cell response in endometrial cancer [56, 57], colorectal cancer [56], meningiomas [58], and high-grade gliomas [33] with an increased number of CD8+ tumor-infiltrating lymphocytes. Here, an increased density of total CD3+ T lymphocytes and/or CD4+ or CD8+ subsets in spatial patterns suggesting interaction with the tumor microenvironment was observed in eight of nine (89%) *POLE*-mutated primary glioblastomas or spinal metastases compared to *POLE* WT controls. The T cell response in tumors with defective polymerase proofreading may be due to an enhanced neoantigen load as a consequence of hypermutation, and contribute to a favorable outcome in endometrial cancer [56, 59, 60]. Similarly, the mean overall survival of 21 months observed here in *POLE/POLD1* germline variant carriers with a glioblastoma compares favorably with the median overall survival of 8 months for glioblastomas according to a recent CBTRUS Statistical Report [61].

Treatment with ICIs does not generally improve survival of glioblastoma patients, e.g. as shown in a randomized phase 3 clinical trial on the effect of programmed death-1 (PD-1) inhibitor nivolumab versus anti-VEGF antibody bevacizumab in patients with recurrent glioblastoma [62]. However, a selected group of glioma patients may respond to ICIs. These may be patients with tumors harboring high mutation and neoantigen loads due to defective MMR or polymerase proofreading, as shown for patients with colorectal cancer [12, 63]. For example, there are reports of durable response to nivolumab in two siblings with recurrent glioblastoma carrying a biallelic *PMS2* germline variant [40], and of clinical and immunologic response to pembrolizumab in a patient with hypermutated glioblastomas and a heterozygous *POLE* germline variant affecting the exonuclease domain [31]. Immunotherapy benefits have also been described in patients with tumors harboring *POLE* variants outside of the exonuclease domain [64]. Therefore, all *POLE/POLD1* germline variant carriers with gliomas showing features of defective polymerase proofreading, i.e. 87% of gliomas analyzed here, may benefit from immunotherapy. Determining TMB and reporting the data in routine diagnostics of gliomas may be an option to identify such patients.

A homozygous *Pold1* knockout in mice leads to peri-implantation lethality [65]. In line with these findings, no LN-229 glioblastoma cell clones devoid of WT *POLD1* alleles could be generated here. In the single viable LN-229 glioblastoma clone with a *POLE* knockout, a mutator phenotype was observed. Similarly, a *POLE* knockout in human near-haploid HAP1 cells was found to promote mutagenesis [66]. In *POLE* knockout cells, DNA polymerase δ may take over replication of the leading DNA strand normally replicated by DNA polymerase ε [10], but with lower fidelity than DNA polymerase ε [10], possibly explaining the increased mutation rate. This is the case because DNA polymerase δ may play a role in replication of both the leading and lagging DNA strands [67]. In contrast, the fact that no viable *POLD1* knockout LN-229 glioblastoma cell clone could be generated here suggests that there is no sufficient compensation by DNA polymerase ε in this cellular model. Furthermore, delayed S phase progression was detected in the *POLE* knockout LN-229 glioblastoma clone indicative of impaired polymerase function. Similarly, impaired S phase progression was detected in fibroblasts from patients with biallelic *POLE* variants and IMAGEI syndrome [68]. Our data suggest that DNA polymerase δ function is essential in glioblastoma cells, and that loss of *POLE* impacts mutation rate, cell cycle progression, and glioblastoma cell survival. While it was our intention to model *POLE* deficiency in glioblastoma cells by generating a *POLE* knockout LN-229 clone, this cell clone is not an ideal model for the heterozygous *POLE* missense variants detected here in glioma patients.

The data compiled here support the notion that brain tumor risk and skin anomalies may be associated, implying that certain skin lesions may point to increased brain tumor risk [69]. In the 37 brain tumor patients with *POLE* or *POLD1* germline variants summarized in this study (references are listed in the Additional file 1: Table S6), these were pilomatricomas, fibromas, and particularly café-au-lait macules, also observed in a colorectal cancer patient with a *POLE* germline variant [70], and one-third of *POLE*-deficient individuals affected by IMAGEI syndrome without cancer [68]. Thus, PPAP has similarities with neurocutaneous disorders, e.g. neurofibromatosis type 1 and type 2, and tuberous sclerosis, which are characterized by combined brain tumor risk and presence of hyper- or hypopigmented cutaneous lesions [71–73], and with hereditary non-polyposis colorectal cancer, which is characterized by an increased risk of colorectal, brain, sebaceous gland tumors, and keratoacanthomas [74], and mimics constitutional MMR deficiency associated with a combination of very similar features [75].

## Conclusion

Our data provide evidence that deleterious *POLE* and *POLD1* germline missense variants predispose to gliomas, which should be considered a part of the PPAP tumor spectrum, and increase the risk of spinal metastasis development. These findings await confirmation in studies on larger cohorts of glioma families. In most gliomas from patients carrying *POLE/POLD1* germline variants, features of defective polymerase proofreading, e.g. hypermutation, *POLE/POLD1*-associated mutational signatures, multinucleated cells, and increased intratumoral T cell response, were observed, potentially rendering these tumors sensitive to ICIs. Data compiled here suggest that giant cell glioblastomas and oligodendrogliomas are overrepresented, cutaneous anomalies, such as café-au-lait macules and pilomatricomas, may serve as clinical markers, and that surveillance colonoscopy is warranted in brain tumor patients carrying *POLE*/*POLD1* germline variants. Genetic testing to assess *POLE/POLD1* variants should be considered in glioma patients with a positive family history of gliomas, other brain tumors, colorectal, endometrial or breast cancer, particularly if they are affected by giant cell glioblastoma or oligodendroglioma, exhibit spinal metastasis or skin lesions, or have a positive history of colorectal adenomas or carcinomas.

### Electronic supplementary material

Below is the link to the electronic supplementary material.

Additional file 1: Supplementary tables

Additional file 2: Supplementary figures

## Data Availability

For reasons of confidentiality, the raw whole-exome sequencing data from the patients cannot be shared. The remaining data generated or analyzed in this study are included in this published article and its supplementary information files.
