# Supplementary material for: Rare germline variants in POLE and POLD1 encoding the catalytic subunits of DNA polymerases ε and δ in glioma families
Source: Acta Neuropathol Commun. 2023 Nov 21;11:184. doi: 10.1186/s40478-023-01689-5 (PMC10664377; doi:10.1186/s40478-023-01689-5)
Supplement: Supplementary file 1 — Additional file 1: Supplementary tables [file 40478_2023_1689_MOESM1_ESM.pdf]

SUPPLEMENTARY TABLES

Rare germline variants in *POLE* and *POLD1* encoding the catalytic subunits of DNA polymerases ε and δ in glioma families

Christine A. M. Weber[1], Nicole Krönke[2], Valery Volk[2], Bernd Auber[1], Alisa Förster[1], Detlef Trost[3], Robert Geffers[4], Majid Esmaeilzadeh[5], Michael Lalk[6], Arya Nabavi[6], Amir Samii[7], Joachim K. Krauss[5], Friedrich Feuerhake[2,8], Christian Hartmann[2], Bettina Wiese[5,9], Frank Brand[1]*, Ruthild G. Weber[1]*

[1]Department of Human Genetics, Hannover Medical School, Hannover, Germany

[2]Department of Neuropathology, Institute of Pathology, Hannover Medical School, Hannover, Germany

[3]Laboratoire CERBA, Saint-Ouen l'Aumône, France

[4]Genome Analytics Research Group, Helmholtz Centre for Infection Research, Braunschweig, Germany

[5]Department of Neurosurgery, Hannover Medical School, Hannover, Germany

[6]Department of Neurosurgery, KRH Klinikum Nordstadt, Hannover, Germany

[7]Department of Neurosurgery, International Neuroscience Institute, Hannover, Germany

[8]Institute for Neuropathology, University Clinic Freiburg, Freiburg, Germany

[9]Department of Neurology, Henriettenstift, Diakovere Krankenhaus gGmbH, Hannover, Germany

Correspondence to: Ruthild G. Weber, M.D., Department of Human Genetics OE 6300, Hannover Medical School, Carl-Neuberg-Str. 1, 30625 Hannover, Germany, Phone +49 511 532 7751, Fax +49 511 532 18520, Email: Weber.Ruthild@mh-hannover.de

Short running title: *POLE* and *POLD1* germline variants in glioma families

*Frank Brand and Ruthild G. Weber have contributed equally as senior authors to this work

Table S1 Analysis of whole-exome sequencing data on leukocyte DNA of two glioma patients (III.1/M1, II.2) of tumor family Fam011 using a linkage-based strategy to identify glioma-predisposing genes

Filtering steps	Number of variants
Total variants in exomes obtained on leukocyte DNA	Patient III.1/M1: 49,958<br>Patient II.2: 49,020
Linkage-based strategy: high quality variants (read depth ≥20, call quality ≥50, allele fraction ≥30% in all samples) shared by glioma patients III.1/M1 and II.2 are retained	30,093
Rare variants (MAF ≤0.01 in the 1000 Genomes Project, ExAC and gnomAD datasets, and the NHLBI ESP exomes)[a] are retained	1,064
Non-silent variants, i.e. splice site (up to two bases into intron), frameshift, in-frame indels, stop gained/lost and non-synonymous missense) variants, are retained	377
Comparison with identically generated exome data of unrelated in-house control individuals (n=148), variants not present in controls are retained	86
Variants predicted to be deleterious by at least one prediction tool (MutationTaster, SIFT, PolyPhen-2, PROVEAN)[b] are retained	65
Variants in cancer predisposing genes (Rahman 2014), and verified by targeted sequencing to co-segregate with the glioma phenotype are retained	1 (*POLE*)

MAF, minor allele frequency

[a]1000 Genomes Project data (https://www.internationalgenome.org), Exome Aggregation Consortium (ExAC) and Genome Aggregation Database (gnomAD) (https://gnomad.broadinstitute.org), National Heart, Lung, and Blood Institute (NHLBI) Exome Sequencing Project (ESP) (https://evs.gs.washington.edu/EVS/)

[b]MutationTaster (https://www.mutationtaster.org), SIFT (https://sift.bii.a-star.edu.sg), PolyPhen-2 (http://genetics.bwh.harvard.edu/pph2/), PROVEAN (https://www.jcvi.org/research/provean)

Table S2 Oligonucleotides used for different applications, as indicated

**Designation**	**Primer sequence (5' to 3')**
Amplification and targeted sequencing of human *POLE* (NM_006231.4)	
POLE_E1_F	CGGAGCGCGCCTCTTGATG
POLE_E1_R	CAGTCAGGCGCGGCGAGTG
POLE_E2_F	AGAAGCAGCAGCAGGTGGCATTA
POLE_E2_R	ACGCTATGACCAGAAGGGTTGC
POLE_E3_F	GTGCTGAGTTTCCCGAAAGGT
POLE_E3_R	TGCACTGGAAGCCTCCCAT
POLE_E4+E5_F	GGCTGTTCATGGGATATAGGAGC
POLE_E4+E5_R	CCATCACCCAACAGATGACCTG
POLE_E6_F	TGAGTGTTTACCCTCTCACCCA
POLE_E6_R	TGCTACGTGTTCCTGTCTCC
POLE_E7_F	CCTACTGAGTTGGAACTCTGGTC
POLE_E7_R	CCAGGAAAGTCTATTCTTCTGTGG
POLE_E8_F	CAGCCAGGAGATGATCATTATGGGTG
POLE_E8_R	GAGTCAGATTCACTCTCCAGCACTG
POLE_E9_F	ACCAGAGGGAGGTAGAGCAG
POLE_E9_R	CTAACAGTGGGGCAGATGCTG
POLE_E10_F	GCTGTGGACTTCTTTGTAGTGAGA
POLE_E10_R	CTGATCTGACGGAATGCCTGAG
POLE_E11_F	CATGAGGCTGCTGCTTCTGAAC
POLE_E11_R	GAGCCACCTCCTAAGTCGACA
POLE_E12_F	ACCTCCCGTGTCTGGGTTC
POLE_E12_R	ATGTGGTGACAGCACAGTCTGC
POLE_E13_F	GTGGTGCCCAGTTTTGCCAG
POLE_E13_R	GGAGCGGGCTGGCATACATG
POLE_E14_F	CCTGTGCCGGTCTCCTTACT
POLE_E14_R	CAGCACTCCTGGGACATCCA
POLE_E15_F	GCAGCCATTTACCACGAGGT
POLE_E15_R	AGAAGCCACACCCGGTGAG
POLE_E16_F	CTTCCGGAGCTTTCTCGGGC
POLE_E16_R	CCTGTGTCATCCGTCCACAGC
POLE_E17+E18_F	AAGGGGTTGGTGAACTGCAC
POLE_E17+E18_R	GCTTCCCACCAAGTGGAGA
POLE_E19_F	GGCCGCCTTTCTCCACTTGG
POLE_E19_R	ATGGACCAACGCAGCCCAG
POLE_E20_F	GCAGTGTATCTGAGCTGTTGC
POLE_E20_R	AGAGGATCCCAGGCAAGGA
POLE_E21_F	GGCCAGTCTAGGAGAAGAATGTAC
POLE_E21_R	TCCCTCCAACATTCCTTGAATCAG
POLE_E22_F	TCCCTTGTCCCTGTCGCATC
POLE_E22_R	AGGAGCAAGGTCGTGAGTTCC
POLE_E23_F	GTCCTGGGGGCAAGCATGG

POLE_E23_R	GTGCTGCAGAGCCAGTGACA
POLE_E24_F	CATCCACTCCTGGAGGGACAC
POLE_E24_R	CCTGGCTCCTGATCCAACCTC
POLE_E25_F	CTCGTGGATGACAGGTGGAG
POLE_E25_R	GTTCTTCGGTCACCTTGGTCTC
POLE_E26_F	TCTGCGTCTCAGGCTAGAGA
POLE_E26_R	CAGCCCAAATCTGTAAGGAACC
POLE_E27_F	CAGCTGTTACACGTCCTTATTTCC
POLE_E27_R	AAGTCAAGAGTGAAGACGCCAG
POLE_E28+E29_F	GCCCTGTGCTCAGCATGAA
POLE_E28+E29_R	GCTTCACTACAGCACACACAG
POLE_E30_F	GCAGCCCGAGATCCTGAGA
POLE_E30_R	CCTAGGGGTCAGGACGCATC
POLE_E31_F	CTAGGGAGGCTGCTTGTGAGA
POLE_E31_R	ACTCCAGGCCCACTCTAACC
POLE_E32_F	GGAGTTTCCTGCCCATCGTAG
POLE_E32_R	GGAGGCCAGGCTAGATCATG
POLE_E33_F	GCACCAGAAGTCTAGGACTCCG
POLE_E33_R	GTCCACTGTCGCTGCTGTTC
POLE_E34+E35_F	GTGTGTCTGCCAAAAGTGGC
POLE_E34+E35_R	CCTTGAGGACAAGACCTGGAG
POLE_E36_F	GTCGTGATTGAATTGGCAGTGC
POLE_E36_R	CAGCTGGACTGAACAGCGAT
POLE_E37_F	CGCCTACAAGGTGAGAGTGG
POLE_E37_R	CATCGCCGGGTCACAGAGA
POLE_E38_F	ATGAGCAGGTGAGCACAGTA
POLE_E38_R	TCCTCGGCACTATTGCCTTG
POLE_E39_F	TTGCTGGTTCTGGAGGTGGT
POLE_E39_R	CCAATGGACCCTGTCTTAGACC
POLE_E40_F	GCCGTGTAGGGAAAACCTGTG
POLE_E40_R	CTTATCCCAGAGGCACTGGCT
POLE_E41_F	TTCTGCTTCAGGACCCTCAG
POLE_E41_R	TCCTGCACTTCTAACGACCTC
POLE_E42_F	CCAGGAAGCCTGAGGGATGATG
POLE_E42_R	TGCAGTGTCTGCTGCTCACG
POLE_E43_F	CAGGTTGGAGGAGCCTTCGC
POLE_E43_R	AGGCTCCGCCCGATCTGATT
POLE_E43_F2	CAGCCGCGTTGTCTCATGTGTTAC
POLE_E43_R2	CTACTAACCATGAGTCCCTTCAGTGGG
POLE_E44_F	GAGGCAATCTGACCAGCCAT
POLE_E44_R	ACCACCCATGGCACACAGA
POLE_E45_F	GCCATGTGCCAGTGACCTAA
POLE_E45_R	ACGCATTACAGCCTCACCTTG
POLE_E46_F	AGTTCTCCGAGGAGGCCCAGTT

POLE_E46_R	CTCGGATGTTCTGCTCCACAGTG
POLE_E46_F2	GCCTAAGGTCCAGAGGGTTC
POLE_E46_R2	GCCAATCCATGTGAGTCAGAGG
POLE_E47_F	GCTGCACTCAGGAGACTCGC
POLE_E47_R	GGCCCCTTGGAAGACACCA
POLE_E48+E49_F	TCGAGGCTTACTGATGGGC
POLE_E48+E49_R	GACAGTCAGGGTGGTCAGTG
POLE_E48_R	GCTGAGCCGAGGCAGATGAG
POLE_E49_F	CTGAGTGGACTGGGGTCTCAC
POLE_E49_R	GATGTGGCCTTGGCATCAGG
Amplification and targeted sequencing of human *POLD1* (NM_002691.4)	
POLD1_E1_F	GTGGCCTTGCCCGCACTTG
POLD1_E1_R	TGTGCCTGGCTGCTCAAGC
POLD1_E2_F	CATGGGATGCCATGGAGAC
POLD1_E2_R	TGCAGCTGACTCTTGGACC
POLD1_E3_F	AGAACCACATGCCATCCTG
POLD1_E3_R	CAGCTCTATGGCAACCAGAG
POLD1_E4_F	ACACACCTTGGAGGACCCTGAG
POLD1_E4_R	GGAGGGATGATCAGAGGTGCAGG
POLD1_E5+E6_F	GCTTCGCTCCCTACTTCTACAC
POLD1_E5+E6_R	GTGTCCACCATGAACCTGGAG
POLD1_E7+E8_F	GTACGGCCTCTGCCTCACT
POLD1_E7+E8_R	ACCGGACCTGCCTTTCCGA
POLD1_E9_F	CCTGCTGTGTTGGGAGTGAGG
POLD1_E9_R	AGGAGCTGATGGCTCAGGACG
POLD1_E10_F	GGAAGTAGGGGAATCCGAGGC
POLD1_E10_R	ATGCACCAAGCCATGTGGAGAG
POLD1_E11+E12_F	CCGTTCTTCAGGCTTATGTGACG
POLD1_E11+E12_R	TGAAGTTGAGGTCAGAGGCCG
POLD1_E13+E14_F	AGGCTACCTCACCCTGACC
POLD1_E13+E14_R	CACCTCAGCCTCCCAGAGATTA
POLD1_E15_F	AGGGTGAGGCCACAAGACAG
POLD1_E15_R	CTCTGGGCCTGTGCCTATGC
POLD1_E16_F	AGGACCGTAGGGCAGAGGT
POLD1_E16_R	CAGTGAGGGACAGGGATTGCC
POLD1_E17_F	GCACTCACTTCCAGAAAGGAGCC
POLD1_E17_R	ACATGGAGCCCTGGTCTCCTA
POLD1_E18+E19_F	GCCCACGTTCACTGCACATG
POLD1_E18+E19_R	CCAAAGAACGGGACACCCTGAG
POLD1_E20_F	GGCGTCTCCAGATTGGGGCT
POLD1_E20_R	ACCCCGGGATCTGAGGAGGA
POLD1_E21+E22_F	TGAGACCCGTGGAGGCACCA
POLD1_E21+E22_R	CCGCCCATCTCGGAAAGCAG
POLD1_E23+E24_F	CCCATGACCACCCCGTGTC

POLD1_E23+E24_R	AGCCGATGGGGTGGGTGTCTA
POLD1_E25+E26_F	GGATGGGGTGGCCCAGTTC
POLD1_E25+E26_R	GGTTGGGTCCACAACCCACAG
POLD1_E27_F	GACCAAAGTCCTGGGAACAGCC
POLD1_E27_R	CACATGAACCAGGCAGCACAGGTG
Generation of a *pSpCas9(BB)-2A-GFP-POLE-sgRNA* construct	
sgRNA1_POLE_E2_KO_F	AAACCAGTTTCGGCACTCAAGCGC
sgRNA1_POLE_E2_KO_R	CACCGCGCTTGAGTGCCGAAACTG
Generation of a *pSpCas9(BB)-2A-GFP-POLD1-sgRNA* construct	
sgRNA1_POLD1_E3_KO_F	CACCGCATCAGCCATAGATCCTCGC
sgRNA1_POLD1_E3_KO_R	AAACGCGAGGATCTATGGCTGATGC
Amplification and sequencing of predicted Cas9 exonic off-target sites on DNA of selected human LN-229 cell clones	
POLE_sgRNA1_OT_DAGLB_F	CGAAGAACCCTGGGAAGACC
POLE_sgRNA1_OT_DAGLB_R	CCTTCCGGATCAGCGTTTCTG
POLE_sgRNA1_OT_EHMT1_F	ATCGGGGTGAGGAAGCTTTG
POLE_sgRNA1_OT_EHMT1_R	CTCGTCCTCTAATCGGCGACG

E, exon; F, forward; KO, knockout; OT, off-target; R, reverse; sgRNA, single guide RNA

Table S3 Multimodal assessment of gliomas of patients carrying rare *POLE* or *POLD1* germline variants with respect to burden and signatures of somatic mutations determined in tumor DNA as well as histological and immunological characteristics of tumor sections, i.e. features of defective polymerase proofreading

**Patient ID**	**Gene**	**Amino acid change**	**P/R/M**	**Histology**	**CNS WHO grade**	**TMB ≥17 mut/Mb[a]**	***POLE/POLD1*-assoc. mutational signature[b]**	**Multinucleated cells/enlarged nuclei**	**Increased[c] density of T cells expressing**			**Increased[c] density of cells expressing**	**PPD score[d]**
									**CD3**	**CD4**	**CD8**	**CD68**	
Fam011-III.1/M1	*POLE*	p.(R47W)	P	Glioblastoma	4								2/5
			M	Gliosarcoma	4								4/5
WI70-III.1	*POLE*	p.(R259H)	P	Glioblastoma	4								1/5
WI127-III.2			P	Glioblastoma	4								2/5
WI161-II.1	*POLE*	p.(R260Q)	P	Glioblastoma	4								3/5
WI207-III.1	*POLE*	p.(T457M)	P	Astrocytoma	2	NA	NA		NA	NA	NA	NA	1/1
WI140-III.1	*POLE*	p.(R1082H)	P	Glioblastoma	4								3/5
WI69-III.1	*POLE*	p.(A1420V)	P	Glioblastoma	4								1/5
WI104-III.1	*POLE*	p.(R2165H)	P	Astrocytoma	3				NA	NA	NA	NA	2/3
			R	Astrocytoma	3				NA	NA	NA	NA	1/3
M2[e]	*POLE*	p.(I2255F)	P	Glioblastoma	4								3/5
			M	Gliosarcoma	4								4/5
WI27-III.1	*POLD1*	p.(A145T)	P	Astrocytoma	2				NA	NA	NA	NA	1/3
			R	Astrocytoma	3				NA	NA	NA	NA	0/3
WI40-II.1	*POLD1*	p.(A152V)	P	Oligodendroglioma	2				NA	NA	NA	NA	0/3

Colored box, presence of feature; white box, absence of feature; NA, not analyzed

Assoc., associated; CNS, central nervous system; P, primary tumor; R, recurrent tumor; M, spinal metastasis.
[a]Tumor mutational burden (TMB) of ≥17 mutations per megabase (mut/Mb) was considered as hypermutated, as previously determined for gliomas [Touat et al. 2020]
[b]*POLE*/*POLD1* pathogenic variant, *POLD1* pathogenic variant and mismatch repair deficiency-associated mutational signatures (COSMIC signatures SBS10 or SBS20)
[c]Increased density of T lymphocytes (CD3+, CD4+ or CD8+) or macrophages (CD68+) in *POLE*-mutated primary tumors (glioblastomas) compared to *POLE* WT primary tumors (glioblastomas, n=5) or in *POLE*-mutated spinal metastases compared to a *POLE* WT spinal metastasis
[d]Polymerase proofreading defect (PPD) score: number of features of defective polymerase proofreading to total number of assessed features (an increased number of T lymphocytes was scored with one point, regardless of whether they expressed CD3, CD4, and/or CD8)
[e]The *POLE* variant was detected in the primary tumor and the spinal metastasis of the patient (germline DNA was not available)

Table S4 Details of rare *POLE* and *POLD1* variants identified in this study, including ACMG classification[a] based on our findings and previous observations

Patient ID		Gene	Nucleotide change	Amino acid change	dbSNP[b]	MAF[c]	MAF comparison of study vs. control cohort (*p* value)[d]	PPD score[e]	CADD score[f]	HGMD[g]	ClinVar[h]	ACMG classification[a]
Fam 011-	III.1/ M1	*POLE*	c.139C>T	p.(R47W)	rs143626223	0.001243	0.1428	2/5 (P), 4/5 (M)	27.1	DM?	VUS (10), LB (5)	**LP:** PS3, PP1, PP2, PP3, BS1
	II.2							N/A				
WI70-III.1		*POLE*	c.776G>A	p.(R259H)	rs61732929	0.009734	0.3340	1/5	23.3	DM?	LB (16), B (5)	**VUS:** PS3, PM1, PP1, PP2, PP3, BS1, BP6
WI127-III.2								2/5				
WI161-II.1		*POLE*	c.779G>A	p.(R260Q)	rs5744752	0.0001242	0.0175	3/5	23.8	DM?	LB (5), B (6)	**VUS:** PS3, PS4_M, PM1, PP2, PP3, BS1, BP6
WI207-III.1		*POLE*	c.1370C>T	p.(T457M)	rs878854842	-	0.0028	1/1	26.1	DM	VUS (3)	**LP:** PS3_M, PS4_M, PM2, PP2, PP3
WI140-III.1		*POLE*	c.3245G>A	p.(R1082H)	rs201744227	0.0002486	0.0323	3/5	25.5	DM?	VUS (9), LB (4)	**LP:** PS3, PS4_M, PM1, PP2, PP3, BS1
WI69-III.1		*POLE*	c.4259C>T	p.(A1420V)	rs41561818	0.004536	0.4268	1/5	21.2	-	LB (9), B (9)	**LB:** PS3_M, PP2, PP3, BS1, BP6
WI104-III.1		*POLE*	c.6494G>A	p.(R2165H)	rs5745068	0.003258	0.3298	2/3 (P), 1/3 (R)	25.9	DM?	LB (4), B (13)	**VUS:** PS3, PP2, PP3, BS1, BP6
M2[i]		*POLE*	c.6763A>T	p.(I2255F)	rs73155056	0.005655	0.5002	3/5 (P), 4/5 (M)	22.3	-	VUS (1), LB (8), B (10)	**VUS:** PS3, PP2, PP3, BS1, BP6
WI27-III.1		*POLD1*	c.433G>A	p.(A145T)	rs137953986	0.002778	0.2893	1/3 (P), 0/3 (R)	20.4	-	LB (8), B (7)	**VUS:** PS3_M, PM1, PP2, PP3, BS1, BP6
WI40-II.1		*POLD1*	c.455C>T	p.(A152V)	rs41563714	0.001057	0.1241	0/3	20.5	DM?	VUS (7), LB (4)	**VUS:** PM1, PP2, PP3, BS1
WII-40-II.1		*POLD1*	c.2546G>A	p.(R849H)	rs3218775	0.008146	0.5889	N/A	22.1	DM?	LB (3), B (14)	**LB:** PM1, PP2, PP3, BS1, BP6

Given are all identified rare (minor allele frequency, MAF ≤0.01), non-silent (i.e. splice site, frameshift, in-frame indels, stop gained/lost and non-synonymous missense) variants with a CADD score[f] ≥ 20.0 in the *POLE* or *POLD1* gene. NCBI reference sequence NM_006231.4 (*POLE*) and NM_002691.4 (*POLD1*). B, benign; DM, disease causing mutation; DM?, likely disease causing mutation; LB, likely benign; LP, likely pathogenic; M, spinal metastasis; N/A, not available; P, primary tumor; R, recurrent tumor; VUS, variant of uncertain significance

[a]American College of Medical Genetics and Genomics (ACMG) standards and guidelines for the interpretation of sequence variants according to Richards et al. 2015; PS3 was awarded for variants with a PPD score >1 in the tumor(s) of variant carriers, PS3_M for variants with a PPD score =1 in the tumor(s) of variant carriers, according to our tumor characterization (see Supplementary Table 3)

[b]SNP database ID (https://www.ncbi.nlm.nih.gov/SNP/)

[c]Minor allele frequency (MAF) according to the Genome Aggregation Database (gnomAD) browser v2.1.1, controls, non-Finnish European population (https://gnomad.broadinstitute.org)

[d]Statistical analyses were conducted using MATLAB and Statistics Toolbox Release 2022a (The MathWorks, Natick, MA) and Fisher's exact test (2-tailed), whereby *p* values <0.05 were considered significant.

[e]Polymerase proofreading defect (PPD) score: number of features of defective polymerase proofreading to total number of assessed features (an increased number of T lymphocytes was scored with one point, regardless of whether they expressed CD3, CD4, and/or CD8) based on our multimodal assessment in tumors of variant carriers (see Supplementary Table 3)

[f]According to CADD (https://cadd.gs.washington.edu)

[g]According to HGMD (https://www.hgmd.cf.ac.uk/ac/index.php)

[h]According to ClinVar (https://www.ncbi.nlm.nih.gov/clinvar/)

[i]Only tumor DNA of the patient was analyzed (non-neoplastic DNA was not available)

Table S5 Genotypes of CRISPR/Cas9-edited cell clones

Target gene	Cell line	Number of cell clones			
		Total	With only wildtype alleles	With a mutant allele	Devoid of wildtype alleles
POLE	LN-229	48	46 (95.8%)	1 (2.1%)	1 (2.1%)
	HCT116	37	37 (100%)	0 (0%)	0 (0%)
POLD1	LN-229	85	65 (76.5%)	20 (23.5%)	0 (0%)
	HCT116	45	45 (100%)	0 (0%)	0 (0%)

Note that LN-229 glioblastoma cells probably harbor more than two copies of *POLE* and *POLD1*

Table S6 Phenotype spectrum of 37 brain tumor patients carrying a rare *POLE* or *POLD1* germline variant reported in this study or previously

Reference	Number of variant carriers	Nucleotide change	Amino acid change	Gen-der	Brain tumor					Additional features (age at diagnosis in years)
					Age at diagnosis (years)	Histology	CNS WHO grade	Molecular characteristics	Localization	
**Brain tumor patients carrying a rare *POLE* germline variant**										
This study	2	c.139C>T	p.(R47W)	F	51	Glioblastoma	4	IDH-WT	Right insula	-
					53	Gliosarcoma	4	IDH-WT	Spinal cord	
				F	62	Astrocytoma	3	NA	Left frontotemporal	-
					65	Glioblastoma	4	NA	Left frontotemporal	
This study	2	c.776G>A	p.(R259H)	M	75	Glioblastoma	4	IDH-WT	Right frontoparietal	-
				M	45	Glioblastoma	4	IDH-WT	Left frontoparietal	-
This study	1	c.779G>A	p.(R260Q)	M	67	Glioblastoma	4	IDH-WT	Left frontal	-
Rosner et al. 2018	1	c.830A>G	p.(E277G)	F	23	Oligodendroglioma	NA	NA	NA	Multiple colorectal adenomas (18 y), endometrial cancer (30 y), café-au-lait macules
Michaeli et al. 2022	1			M	4.5	Medulloblastoma	4	SHH-activated, *TP53*-WT	Posterior fossa	Multiple café-au-lait macules
Sehested et al. 2022	1	c.890C>T	p.(S297F)	M	29	Glioblastoma	4	IDH-WT	Close to the motor-sensory cortex	Colorectal cancer (20 y), multiple colorectal adenomas (20y), pilomatricoma (28 y), multiple café-au-lait macules (30 y), several skin papules and cysts (30 y)
Galati et al. 2020	1			NA	30	Glioblastoma	4	NA	NA	-
Rohlin et al. 2014	2	c.1089C>A	p.(N363K)	M	28	Giant cell glioblastoma	4	NA	NA	-
Palles et al. 2022				F	35	Brain tumor	NA	NA	NA	-
Vande Perre et al. 2019	3			F	30	Giant cell glioblastoma	4	IDH-WT	Right pedunculo-thalamo-pituitary, including caudate nucleus	Colonic polyps (19 y), colorectal cancer (23 y)

				M	45	Giant cell glioblastoma	4	IDH-WT	Right frontal	Colorectal cancer (41 y)
				F	52	Giant cell glioblastoma	4	IDH-WT	Right temporal	Rectal adenocarcinoma (37 y), colonic polyps
Hamzaoui et al. 2020	1	c.1102G>A	p.(D368N)	M	16	Glioblastoma	4	NA	NA	Pilomatricoma (23 y)
Palles et al. 2013; Palles et al. 2022	1	c.1270C>G	p.(L424V)	F	61	Glioblastoma	4	NA	NA	Colorectal cancer (40 y), colonic adenomas (40 y)
Valle et al. 2014, Bellido et al. 2016	1			F	30	Oligodendroglioma	3	NA	NA	Colorectal cancer (28 y), colonic polyps (28 y)
Spier et al. 2015	1			M	47	Glioblastoma	4	NA	NA	Colorectal cancer (27 y), colorectal adenomas (27 y), fibromas, neuroendocrine carcinoma of the ascending colon (41 y)
Elsayed et al. 2015	1			M	15	Astrocytoma	NA	NA	NA	Cecum adenocarcinoma (30 y)
Johanns et al. 2016	1			M	31	Glioblastoma	4	IDH-WT	Left frontotemporal	Colonic polyps, two spinal glioblastoma metastases
Hamzaoui et al. 2020	1			M	66	Glioblastoma	4	NA	NA	Multiple colonic adenomas (50 y)
Galati et al. 2020	1			NA	30	Glioblastoma	4	NA	NA	-
Dodgshun et al. 2020	1	c.1307C>G	p.(P436R)	F	17	Astrocytoma	3	IDH-mutated	Hemispheric	-
Galati et al. 2020	1			NA	17	Astrocytoma	NA	NA	NA	-
Sehested et al. 2022	1	c.1331T>A	p.(M444L)	F	11	Glioma	NA	NA	Tectal plate	Colorectal cancer (13 y), numerous adenomatous polyps (13 y), multiple café-au-lait macules
Lindsay et al. 2019	1	c.1366G>C	p.(A456P)	F	5	Medulloblastoma	NA	Non-WNT, non-SHH	Left cerebellar	Café-au-lait macules, pilomatricomas, renal cyst, osteochondroma
This study	1	c.1370C>T	p.(T457M)	M	35	Astrocytoma	2	IDH-mutated	Right parietal	-

Sehested et al. 2022	1	c.1381T>A	p.(S461T)	F	4	Medulloblastoma	4	SHH A subgroup with somatic *TP53* mutation	Right cerebellar hemisphere	Multiple café-au-lait macules
Barresi et al. 2019	1	c.2224C>T	p.(R742C)	M	39	Giant cell glioblastoma	4	IDH-WT	Frontal	-
This study	1	c.3245G>A	p.(R1082H)	M	58	Glioblastoma	4	IDH-WT	Left basal ganglia	-
This study	1	c.4259C>T	p.(A1420V)	F	57	Glioblastoma	4	IDH-WT	Left temporal	Invasive ductal breast cancer (53 y)
This study	1	c.6494G>A	p.(R2165H)	M	23	Astrocytoma	3	NA	Left frontal	Café-au-lait macules
					34	Astrocytoma	3	IDH-mutated	Left frontal	
**Brain tumor patients carrying a rare *POLD1* germline variant**										
This study	1	c.433G>A	p.(A145T)	F	28	Astrocytoma	2	-	Right central	-
					36	Astrocytoma	3	IDH-mutated	Right frontal	
This study	1	c.455C>T	p.(A152V)	M	29	Oligodendroglioma	2	IDH-mutated, 1p/19q-codeleted	Left frontal	-
Jacobs et al. 2018	1	c.1361G>A	p.(R454H)	M	65	Oligodendroglioma	2	IDH-mutated, 1p/19q-codeleted	NA	-
Palles et al. 2013	1	c.1433G>A	p.(S478N)	M	26	Astrocytoma	NA	NA	NA	Colonic adenomas/polyps (33 y)
This study	1	c.2546G>A	p.(R849H)	F	34	Oligodendroglioma	3	IDH-mutated, 1p/19q-codeleted	Right frontal	-

NCBI reference sequence NM_006231.4 (*POLE*) and NM_002691.4 (*POLD1*).
CNS, central nervous system; F, female; M, male; NA, not available; WHO, World Health Organization; WT, wildtype.

References

Barresi V, Simbolo M, Mafficini A, Piredda ML, Caffo M, Cardali SM, Germanò A, Cingarlini S, Ghimenton C, Scarpa A (2019) Ultra-mutation in IDH wild-type glioblastomas of patients younger than 55 years is associated with defective mismatch repair, microsatellite instability, and giant cell enrichment. Cancers (Basel) 11:1279. https://doi.org/10.3390/cancers11091279

Bellido F, Pineda M, Aiza G, Valdés-Mas R, Navarro M, Puente DA, Pons T, González S, Iglesias S, Darder E, Piñol V, Soto JL, Valencia A, Blanco I, Urioste M, Brunet J, Lázaro C, Capellá G, Puente XS, Valle L (2016) POLE and POLD1 mutations in 529 kindred with familial colorectal cancer and/or polyposis: review of reported cases and recommendations for genetic testing and surveillance. Genet Med 18:325-332. https://doi.org/10.1038/gim.2015.75

Dodgshun AJ, Fukuoka K, Edwards M, Bianchi VJ, Das A, Sexton-Oates A, Larouche V, Vanan MI, Lindhorst S, Yalon M, Mason G, Crooks B, Constantini S, Massimino M, Chiaravalli S, Ramdas J, Mason W, Ashraf S, Farah R, Van Damme A, Opocher E, Hamid SA, Ziegler DS, Samuel D, Cole KA, Tomboc P, Stearns D, Thomas GA, Lossos A, Sullivan M, Hansford JR, Mackay A, Jones C, Jones DTW, Ramaswamy V, Hawkins C, Bouffet E, Tabori U (2020) Germline-driven replication repair-deficient high-grade gliomas exhibit unique hypomethylation patterns. Acta Neuropathol 140:765-776. https://doi.org/10.1007/s00401-020-02209-8

Elsayed FA, Kets CM, Ruano D, van den Akker B, Mensenkamp AR, Schrumpf M, Nielsen M, Wijnen JT, Tops CM, Ligtenberg MJ, Vasen HF, Hes FJ, Morreau H, van Wezel T (2015) Germline variants in POLE are associated with early onset mismatch repair deficient colorectal cancer. Eur J Hum Genet 23:1080-1084. https://doi.org/10.1038/ejhg.2014.242

Galati MA, Hodel KP, Gams MS, Sudhaman S, Bridge T, Zahurancik WJ, Ungerleider NA, Park VS, Ercan AB, Joksimovic L, Siddiqui I, Siddaway R, Edwards M, de Borja R, Elshaer D, Chung J, Forster VJ, Nunes NM, Aronson M, Wang X, Ramdas J, Seeley A, Sarosiek T, Dunn GP, Byrd JN, Mordechai O, Durno C, Martin A, Shlien A, Bouffet E, Suo Z,

Jackson JG, Hawkins CE, Guidos CJ, Pursell ZF, Tabori U (2020) Cancers from novel Pole-mutant mouse models provide insights into polymerase-mediated hypermutagenesis and immune checkpoint blockade. Cancer Res 80:5606-5618. https://doi.org/10.1158/0008-5472.Can-20-0624

Hamzaoui N, Alarcon F, Leulliot N, Guimbaud R, Buecher B, Colas C, Corsini C, Nuel G, Terris B, Laurent-Puig P, Chaussade S, Dhooge M, Madru C, Clauser E (2020) Genetic, structural, and functional characterization of POLE polymerase proofreading variants allows cancer risk prediction. Genet Med 22:1533-1541. https://doi.org/10.1038/s41436-020-0828-z

Jacobs DI, Fukumura K, Bainbridge MN, Armstrong GN, Tsavachidis S, Gu X, Doddapaneni HV, Hu J, Jayaseelan JC, Muzny DM, Huse JT, Bondy ML (2018) Elucidating the molecular pathogenesis of glioma: integrated germline and somatic profiling of a familial glioma case series. Neuro Oncol 20:1625-1633. https://doi.org/10.1093/neuonc/noy119

Johanns TM, Miller CA, Dorward IG, Tsien C, Chang E, Perry A, Uppaluri R, Ferguson C, Schmidt RE, Dahiya S, Ansstas G, Mardis ER, Dunn GP (2016) Immunogenomics of hypermutated glioblastoma: a patient with germline POLE deficiency treated with checkpoint blockade immunotherapy. Cancer Discov 6:1230-1236. https://doi.org/10.1158/2159-8290.Cd-16-0575

Lindsay H, Scollon S, Reuther J, Voicu H, Rednam SP, Lin FY, Fisher KE, Chintagumpala M, Adesina AM, Parsons DW, Plon SE, Roy A (2019) Germline POLE mutation in a child with hypermutated medulloblastoma and features of constitutional mismatch repair deficiency. Cold Spring Harb Mol Case Stud 5:a004499. https://doi.org/10.1101/mcs.a004499

Michaeli O, Ladany H, Erez A, Shachar SB, Izraeli S, Lidzbarsky G, Basel-Salmon L, Biskup S, Maruvka YE, Toledano H, Goldberg Y (2022) Di-genic inheritance of germline POLE and PMS2 pathogenic variants causes a unique condition associated with pediatric cancer predisposition. Clin Genet 101:442-447. https://doi.org/10.1111/cge.14106

Palles C, Cazier JB, Howarth KM, Domingo E, Jones AM, Broderick P, Kemp Z, Spain SL, Guarino E, Salguero I, Sherborne A, Chubb D, Carvajal-Carmona LG, Ma Y, Kaur K,

Dobbins S, Barclay E, Gorman M, Martin L, Kovac MB, Humphray S, The CORGI Consortium, The WGS500 Consortium, Lucassen A, Holmes CC, Bentley D, Donnelly P, Taylor J, Petridis C, Roylance R, Sawyer EJ, Kerr DJ, Clark S, Grimes J, Kearsey SE, Thomas HJ, McVean G, Houlston RS, Tomlinson I (2013) Germline mutations affecting the proofreading domains of POLE and POLD1 predispose to colorectal adenomas and carcinomas. Nat Genet 45:136-144. https://doi.org/10.1038/ng.2503

Palles C, Martin L, Domingo E, Chegwidden L, McGuire J, Cuthill V, Heitzer E, The CORGI Consortium, Kerr R, Kerr D, Kearsey S, Clark SK, Tomlinson I, Latchford A (2022) The clinical features of polymerase proof-reading associated polyposis (PPAP) and recommendations for patient management. Fam Cancer 21:197-209. https://doi.org/10.1007/s10689-021-00256-y

Rahman N (2014) Realizing the promise of cancer predisposition genes. Nature 505:302-308. https://doi.org/10.1038/nature12981

Richards S, Aziz N, Bale S, Bick D, Das S, Gastier-Foster J, Grody WW, Hegde M, Lyon E, Spector E, Voelkerding K, Rehm HL; ACMG Laboratory Quality Assurance Committee (2015) Standards and guidelines for the interpretation of sequence variants: a joint consensus recommendation of the American College of Medical Genetics and Genomics and the Association for Molecular Pathology. Genet Med 17:405-424. https://doi.org/10.1038/gim.2015.30

Rohlin A, Zagoras T, Nilsson S, Lundstam U, Wahlström J, Hultén L, Martinsson T, Karlsson GB, Nordling M (2014) A mutation in POLE predisposing to a multi-tumour phenotype. Int J Oncol 45:77-81. https://doi.org/10.3892/ijo.2014.2410

Rosner G, Gluck N, Carmi S, Bercovich D, Fliss-Issakov N, Ben-Yehoyada M, Aharon-Caspi S, Kellerman E, Strul H, Shibolet O, Kariv R (2018) POLD1 and POLE gene mutations in Jewish cohorts of early-onset colorectal cancer and of multiple colorectal adenomas. Dis Colon Rectum 61:1073-1079. https://doi.org/10.1097/dcr.0000000000001150

Sehested A, Meade J, Scheie D, Østrup O, Bertelsen B, Misiakou MA, Sarosiek T, Kessler E, Melchior LC, Munch-Petersen HF, Pai RK, Schmuth M, Gottschling H, Zschocke J, Gallon

R, Wimmer K (2022) Constitutional POLE variants causing a phenotype reminiscent of constitutional mismatch repair deficiency. Hum Mutat 43:85-96. https://doi.org/10.1002/humu.24299

Spier I, Holzapfel S, Altmüller J, Zhao B, Horpaopan S, Vogt S, Chen S, Morak M, Raeder S, Kayser K, Stienen D, Adam R, Nürnberg P, Plotz G, Holinski-Feder E, Lifton RP, Thiele H, Hoffmann P, Steinke V, Aretz S (2015) Frequency and phenotypic spectrum of germline mutations in POLE and seven other polymerase genes in 266 patients with colorectal adenomas and carcinomas. Int J Cancer 137:320-331. https://doi.org/10.1002/ijc.29396

Touat M, Li YY, Boynton AN, Spurr LF, Iorgulescu JB, Bohrson CL, Cortes-Ciriano I, Birzu C, Geduldig JE, Pelton K, Lim-Fat MJ, Pal S, Ferrer-Luna R, Ramkissoon SH, Dubois F, Bellamy C, Currimjee N, Bonardi J, Qian K, Ho P, Malinowski S, Taquet L, Jones RE, Shetty A, Chow KH, Sharaf R, Pavlick D, Albacker LA, Younan N, Baldini C, Verreault M, Giry M, Guillerm E, Ammari S, Beuvon F, Mokhtari K, Alentorn A, Dehais C, Houillier C, Laigle-Donadey F, Psimaras D, Lee EQ, Nayak L, McFaline-Figueroa JR, Carpentier A, Cornu P, Capelle L, Mathon B, Barnholtz-Sloan JS, Chakravarti A, Bi WL, Chiocca EA, Fehnel KP, Alexandrescu S, Chi SN, Haas-Kogan D, Batchelor TT, Frampton GM, Alexander BM, Huang RY, Ligon AH, Coulet F, Delattre JY, Hoang-Xuan K, Meredith DM, Santagata S, Duval A, Sanson M, Cherniack AD, Wen PY, Reardon DA, Marabelle A, Park PJ, Idbaih A, Beroukhim R, Bandopadhayay P, Bielle F, Ligon KL (2020) Mechanisms and therapeutic implications of hypermutation in gliomas. Nature 580:517-523. https://doi.org/10.1038/s41586-020-2209-9

Valle L, Hernández-Illán E, Bellido F, Aiza G, Castillejo A, Castillejo MI, Navarro M, Seguí N, Vargas G, Guarinos C, Juarez M, Sanjuán X, Iglesias S, Alenda C, Egoavil C, Segura Á, Juan MJ, Rodriguez-Soler M, Brunet J, González S, Jover R, Lázaro C, Capellá G, Pineda M, Soto JL, Blanco I (2014) New insights into POLE and POLD1 germline mutations in familial colorectal cancer and polyposis. Hum Mol Genet 23:3506-3512. https://doi.org/10.1093/hmg/ddu058

Vande Perre P, Siegfried A, Corsini C, Bonnet D, Toulas C, Hamzaoui N, Selves J, Chipoulet E, Hoffmann JS, Uro-Coste E, Guimbaud R (2019) Germline mutation p.N363K in POLE is associated with an increased risk of colorectal cancer and giant cell glioblastoma. Fam Cancer 18:173-178. https://doi.org/10.1007/s10689-018-0102-6
