# Supplementary material for: Rare germline variants in POLE and POLD1 encoding the catalytic subunits of DNA polymerases ε and δ in glioma families
Source: Acta Neuropathol Commun. 2023 Nov 21;11:184. doi: 10.1186/s40478-023-01689-5 (PMC10664377; doi:10.1186/s40478-023-01689-5)
Supplement: Supplementary file 2 — Additional file 2: Supplementary figures [file 40478_2023_1689_MOESM2_ESM.pdf]

SUPPLEMENTARY FIGURES

Rare germline variants in *POLE* and *POLD1* encoding the catalytic subunits of DNA polymerases ε and δ in glioma families

Christine A. M. Weber[1], Nicole Krönke[2], Valery Volk[2], Bernd Auber[1], Alisa Förster[1], Detlef Trost[3], Robert Geffers[4], Majid Esmaeilzadeh[5], Michael Lalk[6], Arya Nabavi[6], Amir Samii[7], Joachim K. Krauss[5], Friedrich Feuerhake[2,8], Christian Hartmann[2], Bettina Wiese[5,9], Frank Brand[1]*, Ruthild G. Weber[1]*

[1]Department of Human Genetics, Hannover Medical School, Hannover, Germany

[2]Department of Neuropathology, Institute of Pathology, Hannover Medical School, Hannover, Germany

[3]Laboratoire CERBA, Saint-Ouen l'Aumône, France

[4]Genome Analytics Research Group, Helmholtz Centre for Infection Research, Braunschweig, Germany

[5]Department of Neurosurgery, Hannover Medical School, Hannover, Germany

[6]Department of Neurosurgery, KRH Klinikum Nordstadt, Hannover, Germany

[7]Department of Neurosurgery, International Neuroscience Institute, Hannover, Germany

[8]Institute for Neuropathology, University Clinic Freiburg, Freiburg, Germany

[9]Department of Neurology, Henriettenstift, Diakovere Krankenhaus gGmbH, Hannover, Germany

Correspondence to: Ruthild G. Weber, M.D., Department of Human Genetics OE 6300, Hannover Medical School, Carl-Neuberg-Str. 1, 30625 Hannover, Germany, Phone +49 511 532 7751, Fax +49 511 532 18520, Email: Weber.Ruthild@mh-hannover.de

Short running title: *POLE* and *POLD1* germline variants in glioma families

*Frank Brand and Ruthild G. Weber have contributed equally as senior authors to this work

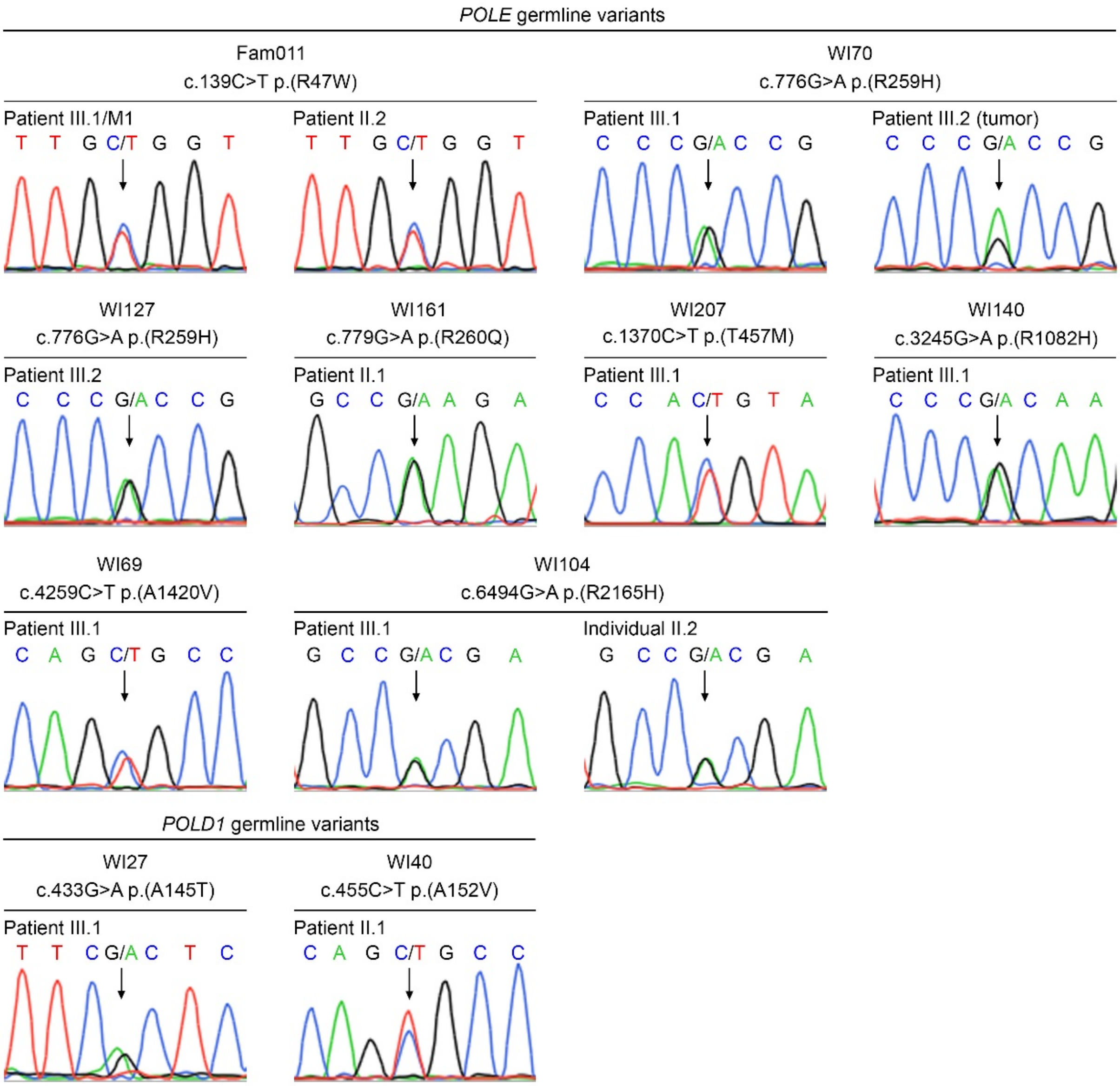

Fig. S1 Electropherograms obtained by targeted sequencing to verify rare non-silent heterozygous *POLE* and *POLD1* variants detected in leukocyte DNA of glioma patients from 10 tumor families by whole-exome sequencing, and to determine segregation within families, if DNA was available. The position of the variants is indicated by arrows. Nucleotide numbering according to NCBI reference sequences: NM_006231.4 (*POLE*) and NM_002691.4 (*POLD1*).

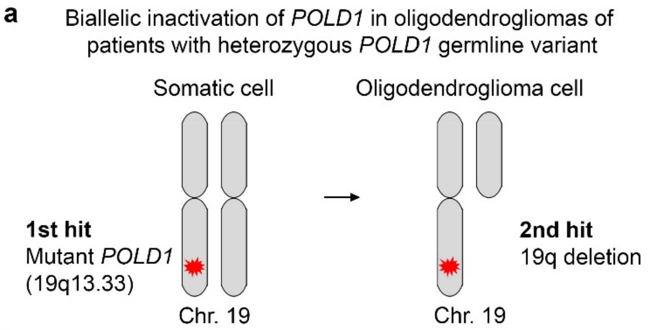

b

	Analyzed patients with 1p/19q-codeleted oligodendrogliomas
POLD1 germline variant	
Carriers	2 (6%)
Non-carriers	32
Total	34

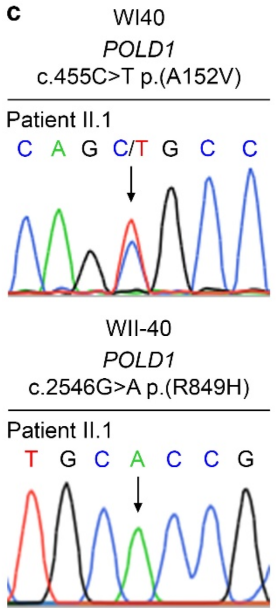

Fig. S2 Rare non-silent *POLD1* germline variants were identified in 6% of patients with 1p/19q-codeleted oligodendrogliomas. **a** Scheme illustrating the hypothesis that *POLD1* germline variants may promote oligodendroglioma development. **b** The presence or absence of *POLD1* germline variants was determined on leukocyte DNA of 34 patients with 1p/19q-codeleted oligodendrogliomas. Given are carriers and non-carriers of rare (minor allele frequency ≤0.01 according to the Genome Aggregation Database browser v2.1.1, controls, non-Finnish European population, https://gnomad.broadinstitute.org) non-silent *POLD1* germline variants predicted to be deleterious by at least one of four prediction tools (MutationTaster, SIFT, PolyPhen-2, PROVEAN) and with a CADD score ≥20. **c** Electropherograms of *POLD1* germline variants in two patients with 1p/19q-codeleted oligodendrogliomas. The heterozygous *POLD1*:c.455C>T p.(A152V) variant was confirmed by targeted sequencing in patient II.1 of tumor family WI40. In a sporadic oligodendroglioma case, patient WII-40-II.1, the homozygous *POLD1*:c.2546G>A p.(R849H) variant was detected by targeted sequencing of all *POLD1* exons. The position of the variants is indicated by black arrows. Nucleotide numbering according to NCBI reference sequence: NM_002691.4. Chr., chromosome.

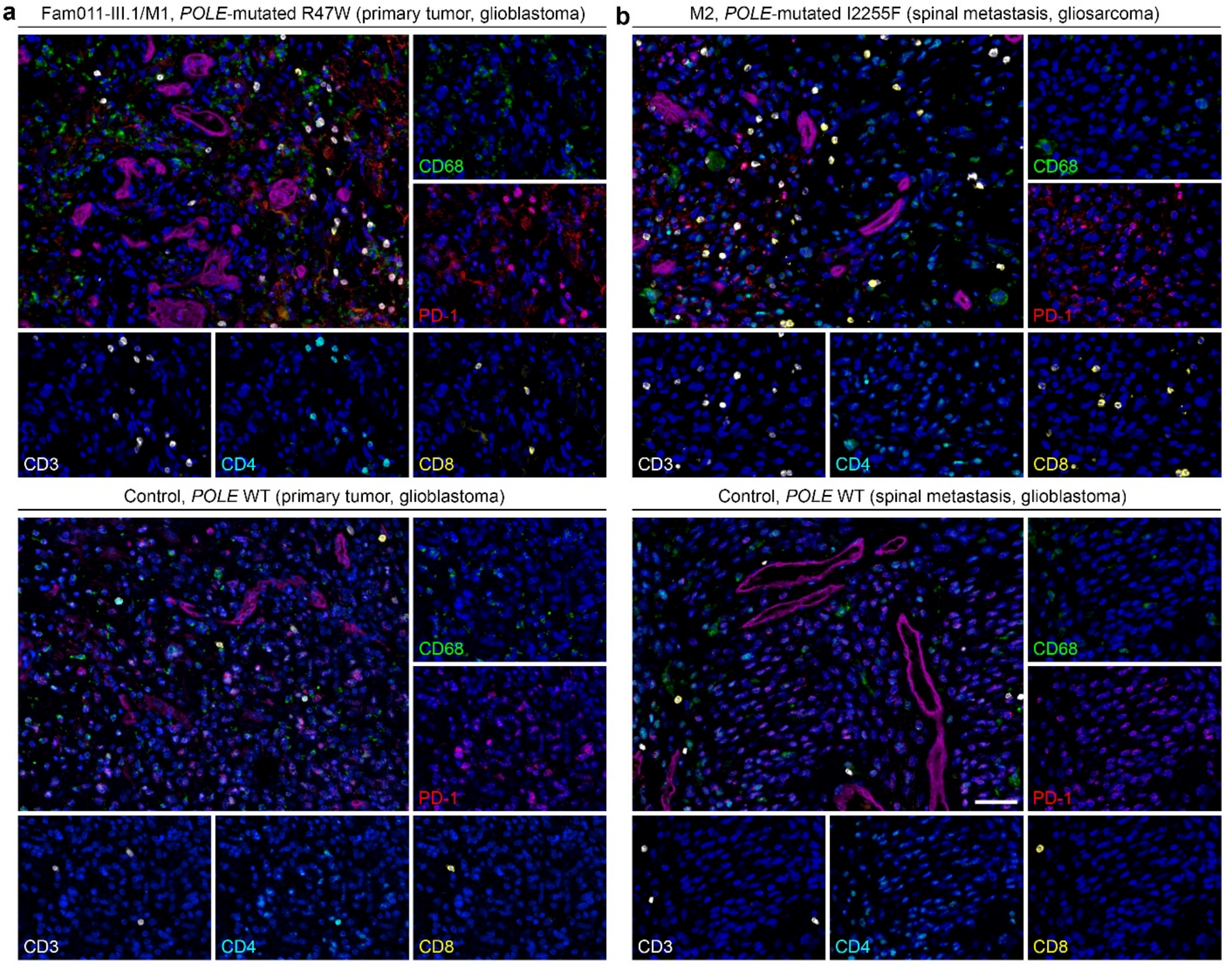

Fig. S3 Immune cell infiltrates were not restricted to the perivascular compartment, but were diffusely distributed in spatial patterns suggesting interaction with the tumor microenvironment, as shown by CD34 co-staining to visualize blood vessels. **a-b** Representative images showing immune cells expressing CD3 (white), CD4 (cyan), CD8 (yellow), CD68 (green), PD-1 (red) in the context of blood vessels (CD34, magenta) in sections of *POLE*-mutated and *POLE* WT glioblastomas (**a**) and spinal metastases (**b**). In images also shown in Fig. 4a, blood vessels were visualized by CD34 co-staining. Nuclei were stained with 4′,6-diamidino-2-phenylindole (DAPI, blue). Scale bar, 50 µm. WT, wildtype.

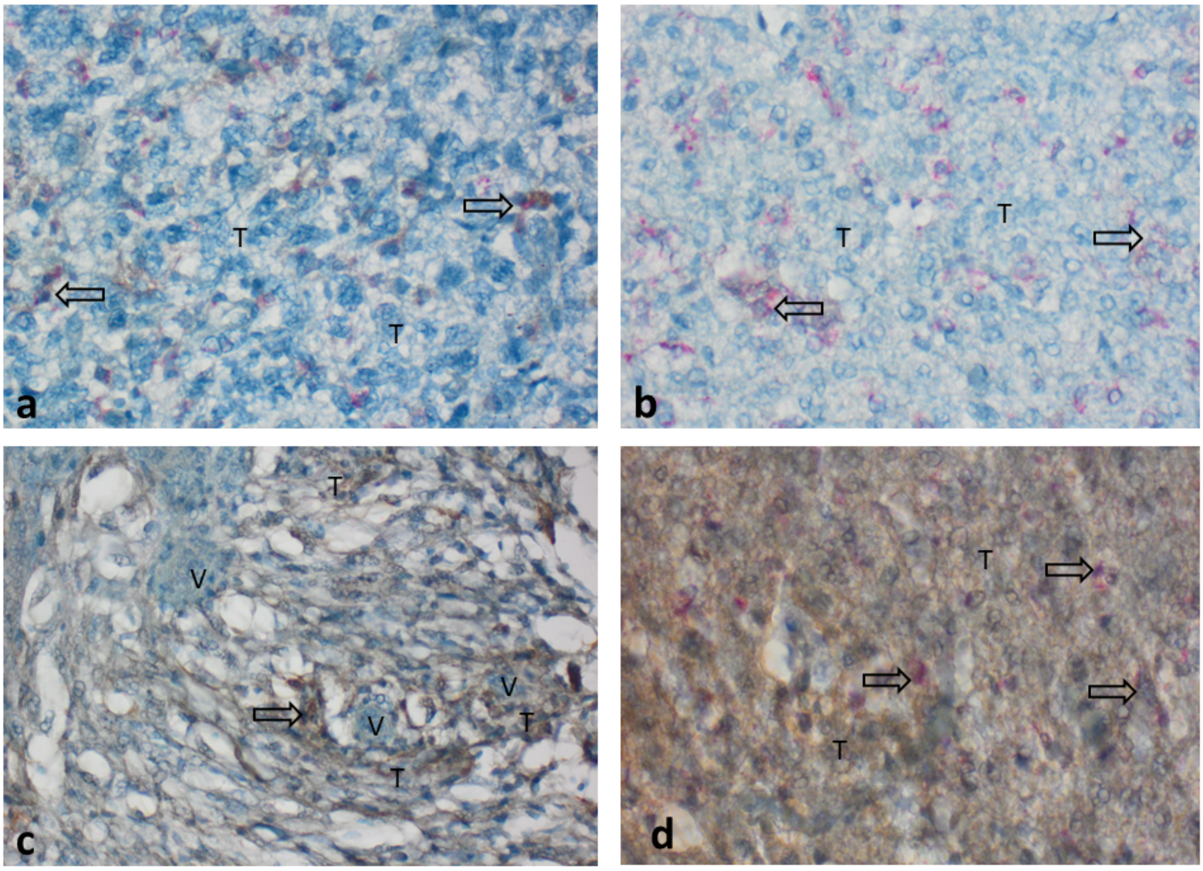

Fig. S4 PD-L1 expression in tumor cells and macrophages by chromogenic duplex immunohistochemistry. Co-expression of PD-L1 (brown) and the macrophage marker CD68 (red) was observed in a subset of glioma-infiltrating macrophages (arrows) throughout all analyzed cases, i.e. seven primary glioblastomas and two spinal metastases from seven patients with rare *POLE* variants. **a-b** Representative examples of cases with PD-L1 expression in less than 30% of glioma cells classified as negative for PD-L1. **c-d** Glioblastomas from patients M2 (**c**) and WI140-III.1 (**d**) showed PD-L1 expression in more than 30% of glioma cells, and were classified as positive for PD-L1. T, solid tumor areas; V, blood vessels/vascular proliferations.
